# Effects of Leydig cell elimination on testicular interstitial cell populations: characterization by scRNA-seq and immunocytochemical techniques

**DOI:** 10.3389/fendo.2024.1423801

**Published:** 2024-08-20

**Authors:** Fu Huang, Jiexia Wang, Hu Wang, Yun Hu, Zhenni Li, Jingfeng Xu, Mengjie Qin, Xin Wen, Shuyan Cao, Xiaoju Guan, Ping Duan, Haolin Chen, Congde Chen

**Affiliations:** ^1^ Key Laboratory of Children Genitourinary Diseases of Wenzhou City, Department of Pediatric Urology, The Second Affiliated Hospital and Yuying Children’s Hospital of Wenzhou Medical University, Wenzhou, Zhejiang, China; ^2^ Key Laboratory of Structural Malformations in Children of Zhejiang Province, Department of Pediatric Urology, The Second Affiliated Hospital and Yuying Children’s Hospital of Wenzhou Medical University, Wenzhou, Zhejiang, China; ^3^ Department of Gynecology and Obstetrics, The Second Affiliated Hospital and Yuying Children’s Hospital of Wenzhou Medical University, Wenzhou, Zhejiang, China; ^4^ Department of Pharmacology, The Second Affiliated Hospital and Yuying Children’s Hospital of Wenzhou Medical University, Wenzhou, Zhejiang, China; ^5^ Zhejiang Provincial Key Laboratory of Anesthesiology, Department of Anesthesiology, The Second Affiliated Hospital and Yuying Children’s Hospital of Wenzhou Medical University, Wenzhou, Zhejiang, China; ^6^ The Basic Medical Research Center of the Second School of Medicine, The Second Affiliated Hospital and Yuying Children’s Hospital of Wenzhou Medical University, Wenzhou, Zhejiang, China

**Keywords:** testicular interstitial cells, rat Leydig cells, scRNA-seq, mesenchymal cells, EDS, hemicastration

## Abstract

**Background:**

The mammalian testicular interstitial cells are not well-defined. The present study characterized the interstitial cell types and their turnover dynamics in adult rats. Additionally, the heterogeneity of the mesenchymal population and the effects of Leydig cell elimination on interstitial homeostasis were further analyzed by scRNA-seq datasets and immunocytochemical techniques.

**Methods:**

Interstitial cells were defined at the transcriptomic level by scRNA-seq and then confirmed and quantified with protein markers. The dividing activity of the major cell types was determined by continuous EdU labeling of the animals for one week. Some of the rats were also treated with a dose of ethylenedimethylsulfonate (EDS) to examine how the loss of Leydig cells (LCs) could affect interstitial homeostasis for three weeks.

**Results:**

Seven interstitial cell types were identified, including cell types (percentage of the whole interstitial population) as follows: Leydig (44.6%), macrophage and dendritic (19.1%), lymphoid (6.2%), vascular endothelial (7.9%), smooth muscle (10.7%), and mesenchymal (11.5%) cells. The EdU experiment indicated that most cell types were dividing at relatively low levels (<9%) except for the mesenchymal cells (MCs, 17.1%). Further analysis of the transcriptome of MCs revealed 4 subgroups with distinct functions, including 1) glutathione metabolism and xenobiotic detoxification, 2) ROS response and AP-1 signaling, 3) extracellular matrix synthesis and binding, and 4) immune response and regulation. Stem LCs (SLCs) are primarily associated with subgroup 3, expressing ARG1 and GAP43. EDS treatment not only eliminated LCs but also increased subgroup 3 and decreased subgroups 1 and 2 of the mesenchymal population. Moreover, EDS treatment increased the division of immune cells by more than tenfold in one week.

**Conclusion:**

Seven interstitial cell types were identified and quantified for rat testis. Many may play more diversified roles than previously realized. The elimination of LCs led to significant changes in MCs and immune cells, indicating the importance of LCs in maintaining testicular interstitial homeostasis.

## Introduction

The mammalian testes have two major functions: spermatogenesis in the seminiferous tubular compartment and steroidogenesis in the interstitial compartment. Spermatogenesis has been extensively studied and well-defined (1, 2), while interstitial cells are far less well-understood (3, 4). In recent years, testicular cells have been studied by the single-cell RNA sequencing (scRNA-seq) technique (5–14). However, most of these studies focused on the seminiferous tubule compartment or the developmental stages. Few addressed the interstitial compartment of the adult testis in depth, partially due to the fact that the number of interstitial cells caught by the technique was usually too low to be analyzed in depth.

In a previous study, we have analyzed the interstitial population of adult rat testes by enriching the interstitial tissue before the scRNA-seq procedure (9). Seven unique interstitial cell types were identified, but the cells were defined based on a mixture of control and EDS-treated samples, so the cell property and transcriptomes for control rats were not defined. Also, the seven cell types identified by RNA tools were not confirmed by protein markers. Since the scRNA-seq technique evaluates cells released from enzymatic digestion of tissues, the cell numbers in the datasets do not always reflect the real numbers *in vivo*. So the true interstitial cell types, numbers, and their turnover dynamics are still not well-established in this species.

Regarding LCs and their stem cells (SLCs), studies found that new LCs can be generated in the interstitial compartment of adult rats after LCs were eliminated by ethylenedimethylsulfonate (EDS) treatment. Although putative SLCs were identified in the interstitial compartment of adult testes (15–20), they were not well-characterized. The SLC markers identified so far all point to the general mesenchymal and peritubular populations. Given the number and heterogeneity of the testicular mesenchymal population, it is still unknown whether all the mesenchymal cells (MCs) have an equal activity to give rise to LCs or there is a special group of stem cells present in this population.

In the present study, we defined the testicular interstitial cell types of untreated adult rats by analyzing a scRNA-seq dataset (9). The cell types identified at the RNA level were confirmed and quantified by protein markers. We also examined how these cells are affected by LC elimination with EDS treatment or by removal of the contralateral testis (hemicastration). The dividing activities of different interstitial cell populations were also defined by continuous labeling of the cells with daily injections of 5-ethynyl-2’-deoxyuridine (EdU) for a week.

## Materials and methods

### Animals

Adult male Sprague-Dawley rats of 60-90 days of age (n=16) were purchased from the Shanghai Animal Centre (Shanghai, China). The animals were maintained in the animal facilities of the Second Affiliated Hospital of Wenzhou Medical University (Wenzhou, China) at 22°C, with a 12-hour light/12-hour dark cycle and free access to water and rat chow. All animal procedures were approved by the Laboratory Animal Ethics Committee of Wenzhou Medical University and were in accordance with the Guide for the Care and Use of Laboratory Animals of NIH (NIH publication #85-23, revised in 1985).

### Treatment of animals

Sixteen rats were randomly separated into 4 groups with an equal number per group. Group one received vehicle administration (DMSO: PBS mixture) plus sham operation to serve as the control. Group two and three were intraperitoneally injected with a single dose (80 mg/kg body weight) of EDS (SKS Chem, Beijing, China, Lot: B9183943), dissolved in a mixture of dimethyl sulfoxide (DMSO): phosphate buffered saline (PBS) (1:3). Group four underwent hemicastration. To label the dividing pool of interstitial cells, all groups were injected with EdU (Thermo-Fisher, Waltham, MA, USA, Cat# C10337) (i.p., 25 mg/kg BW) daily for 7 days from the day on which EDS or hemicastration was carried out. Groups 1, 2, and 4 were killed, and their testes and blood serum were collected at the end of the EdU treatment (1 week after EDS or castration procedure). Group 3 (the second EDS group) was killed, and their testes and blood serum were collected at the end of 8 weeks after EDS administration. The testes were fixed in paraformaldehyde (4%, 6 hrs), and embedded in paraffin for morphological study or frozen for Western blots.

### Preparation of interstitial cell suspensions

The detailed procedures for cell preparation and scRNA-seq can be found in a previous article (9). Briefly, to eliminate contaminations from blood cells, the testicular artery was cannulated and perfused with DMEM/F12 culture medium containing 2.2 g L-1 Hepes, 0.1% BSA, 0.7 g L-1 sodium bicarbonate, pH 7.4. The interstitial tissue and seminiferous tubules of the testes were then separated with fine forceps under a transillumination dissection microscope (21). Interstitial tissue from the testes of each of three animals was combined, and then digested with 1 mg/ml collagenase-IV in DMEM/F12 medium at 34°C for 30 min with slow shaking (90 cycles/min). After allowing the undigested tissue to settle, the dispersed cells were filtered through a 30 µm pore nylon mesh. The cell viabilities, assayed by 0.4% Trypan blue staining, were above 85% for all three experimental groups (control, one and three weeks after EDS treatment).

### Single-cell transcriptomes sequenced by 10X Genomics Chromium

Cell capture, 10x Genomics library preparation, and sequencing were performed by Novogene (Beijing, China). After washing twice in PBS, approximately 10,000 cells were loaded onto 10x Chromium chips with 3′v2 chemistry and barcode to achieve a targeted cell count of 8,000, according to the manufacturer’s instructions (10x Genomics, Pleasanton, CA). After cDNA synthesis, 14 amplification cycles were carried out for each library preparation. The resultant libraries were sequenced using 2×150 paired-end sequencing protocol on an Illumina NovaSeq. 6000 platform (Illumina, San Diego, CA), with a read length of 26bp for the cell barcode and unique molecule identifier (UMI) (read 1), 8bp i7 index read (sample barcode), and 98bp for the actual RNA read (read 2). Each treatment group yielded approximately 550M reads. All downstream single-cell analyses were performed using Cell Ranger and Seurat software.

### Alignment, barcode assignment and UMI counting

For quality control purposes, FastQC was used to perform basic statistics on the quality of the raw reads. Demultiplexed raw sequencing reads were processed and aligned to the rat genome NCBI Rnor6.0 by the 10x Genomics Cell Ranger (v2.1.1) pipeline to generate the filtered gene-barcode matrix containing valid cell barcodes and transcript UMI counts. Only the reads that were confidently mapped to the transcriptome were used for UMI counting. For each gene and each cell barcode, UMIs were counted to construct digital expression matrices, which were filtered a second time using Seurat software with the following two criteria: a gene with expression in more than 3 cells was considered as expressed, and each cell was required to express at least 200 such genes to be counted. Datasets from different treatment groups were integrated using the Cellranger aggr command based on mapped read counts to normalize sequencing depth, producing a single feature-barcode matrix containing all data and clustering models.

### PCA and t-SNE analysis

To reduce the gene expression matrix to its most important features, Cell Ranger uses Principal Components Analysis (PCA) to change the dimensionality of the dataset from cells × genes to cells × M, where M is a user-selectable number of principal components. For visualizing data in 2D space, Cell Ranger passes the PCA-reduced data into t-Stochastic Neighbor Embedding (t-SNE). Cell Ranger uses two different methods for clustering cells by expression similarity, both of which operate in the PCA space: 1) k-means clustering that groups cells into a pre-set number of clusters; and 2) graph-based clustering that builds a nearest-neighbor graph, where cells are linked if they are among the k nearest Euclidean neighbors. It is then followed by Louvain Modularity Optimization, an algorithm which seeks to find highly connected “modules” in the graph20. The Loupe Cell Browser v5.0.0 (10x Genomics) was used to visualize t-SNE projections and “Violin-distributions”.

### Cell type identification and marker exploration

To define the cellular types in detail, the data was projected into t-SNE by the Loupe Browser. Through up-setting K-means from 2 to 10 stepwise, the major clusters for germ cells, immune cells, and non-immune somatic cells were identified. With graph-based clustering, more detailed cell types were defined with the help of published markers for testicular germ and somatic cell populations. According to marker distributions, graph-based sub-clusters were combined and eventually yielded 8 unique cell populations. Upon defining the detailed cell types, the potential markers were identified by the “up-regulated genes per cluster” feature table. The distributions of the top 10 genes were used for heatmap plot. Violin-distributions for representative markers were generated by the Loupe Browser. Gene Ontology (GO) and Kyoto Encyclopedia of Genes and Genomes (KEGG) enrichments of the top 200 DEGs for each cell type were carried out by the online resource of OmicStudio Biotech Inc (Huangzhou, China; https://www.omicstudio.cn/index). GO and KEGG terms with a corrected P-value less than 0.05 were considered significantly enriched within the detectable gene groups.

The raw data in fastq format can be obtained from the National Genomics Data Center/Genome Sequence Archive (NDGC/GSA) repository (https://ngdc.cncb.ac.cn/gsa/browse/CRA004958). The processed expression matrices files can be accessed from the NDGC repository (https://ngdc.cncb.ac.cn/omix/view/OMIX672).

### Tissue preparation and immunohistochemical staining

Paraffin-sections were first processed using the Click-iT procedure to reveal EdU+ cells, followed by immunofluorescent staining of the marker proteins for each interstitial cell type. After washing with PBS for 3 times, the tissue sections were incubated with a first antibody (see **Supplementary Table S1**) overnight at 4°C, washed 3 times with PBS, followed by incubation with a fluorescent dye tagged secondary antibody (see **Supplementary Table S1**) for 45 mins in darkness. Cell nuclei were revealed by DAPI staining. The markers used for labeling each cell types are: Leydig cells (Cytochrome P450 family 11 subfamily a1, CYP11A1), macrophages and dendritic cells (Cluster of differentiation 68, CD68), lymphoid cells (Cluster of differentiation3, CD3), vascular endothelial cells (Cluster of differentiation31, CD31), smooth muscle cells (Actin α2, ACTA2), and mesenchymal cells (Platelet-derived growth factor receptor a, PDGFRA+/CYP11A1-). These markers were selected based on three criteria: 1) Specificity: the gene should be expressed solely by the cell clusters targeted; 2) Universality: the gene should be expressed evenly and universally by the whole cluster to insure a complete coverage of the population; 3) the protein products should have been tested in a similar cell type of testes or other organs (CYP11A1 (22), ACTA2 (20), PDGFRA (23, 24) CD68 (25), CD3 (26, 27), CD31 (28). The three antibodies tested for detecting MC subgroups were ARG1, GAP43 and FN1. The manufacturers and dilutions used for all antibodies are listed in **Supplementary Table S1**.

### Cell quantifications of the tissue sections

Immunofluorescently labelled interstitial cells were counted in each interstitial triangle or quadrangle areas that were covered by squares with an area of 40μm× 40μm up to 80μm× 80μm in size. Also, the total interstitial cells in each triangle/quadrangle area were counted by the number of nuclei presenting in the area. There were about 20 areas counted for each animal that came from at least 5 different sections for each cell type. The percentages of the cell type were calculated by dividing the number of positive cells for each marker protein by the total interstitial cell nuclei counted in the same area. The percentage of each cell type was further normalized based on its proportion in the total interstitial cell types so that the normalized total percentages of the 7 cell types equaled 100%. To analyze the effects of EDS and hemicastration on the relative cell numbers, the percentages of each cell types of the treated rats were divided by the percentages of the cells from the control animals. The ratio conversion was done for each cell type between individual animals across the 2 treatment groups, resulting in 4 independent ratios generated for each cell type.

For the tissues labelled by co-staining of the marker proteins and EdU, the 4 combinational percentages were determined. For Leydig cells as an example, EdU-/CYP11A1-, EdU+/CYP11A1-, EdU-/CYP11A1+, and EdU+/CYP11A1+ cells were counted. The percentages of single and double positive cells were then calculated for each cell type. To analyze the effects of EDS and hemicastration on the cell divisions, the ratios for each cell type were also calculated by dividing the percentages of the treated animals by the percentages of the control rats for each cell type. The conversions were done similarly as for the total cell number (see above). To determine Leydig cell apoptosis, terminal deoxynucleotidyl transferase dUTP nick end labeling (TUNEL) positive and negative cells were counted for CYP11A1+ cells.

Overall, 57,650 cells were counted for the whole experiment. On average, 3,843 cells were counted per animal, and 549 cells were counted for each cell type per animal. The averaged coefficient of variations between animals for each cell types are: LCs (14.43), SMC (13.57), MDC (8.81), TC (13.39), EC (15.60), MC (5.58). The overall coefficient of variations between animals for the whole experiment is 11.90.

### Statistical analyses

Data are expressed as the mean ± standard error of the mean (SEM). For comparisons of two groups, a Student t test was used. For comparisons of multiple groups, ANOVA was applied. The significant differences among individual groups were determined by the LSD test, using SPSS (IBM, Armonk, NY,USA) statistical software package. GraphPad Prism 8.0.1 (GraphPad Software Inc., San Diego, CA,USA) or Excel (Microsoft, Redmond, WA,USA) were used to draw diagrams. Values were considered significant at P<0.05.

## Results

### Interstitial cell heterogeneity defined by the cell transcriptomes

**Figure 1:**
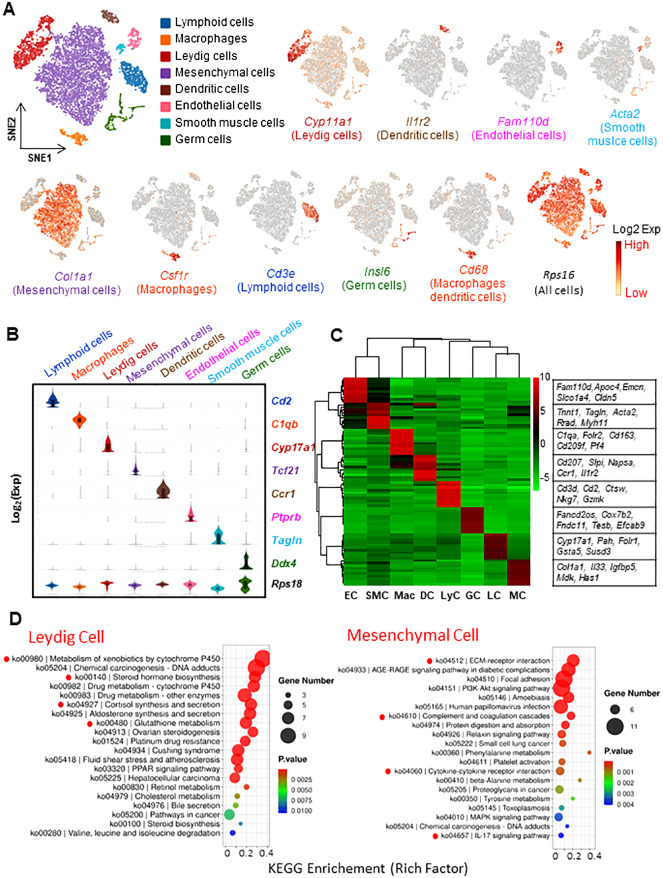
Characterization of interstitial cells of adult rat testis by scRNA-seq technique. **(A)** tSNE plots and clustering analysis of 10,648 interstitial cells. Eight unique cell types were identified based on their transcriptomes. Typical markers for each cell type are also shown. **(B)** Violin-distributions of another set of the marker genes exclusively expressed by each cell type identified. **(C)** Heatmap shows the top 10 marker genes associated with each cell types. **(D)** The top 20 GO or KEGG terms enriched for Leydig and mesenchymal cells. The terms with clear association or significance to the cell characters are highlighted by red dots. tSNE: t-distributed stochastic neighbor embedding. EC, Endothelial Cells; SMC, Smooth Muscle Cells; Mac, Macrophages; DC, Dendritic Cells; LyC, Lymphoid Cells; GC, Germ Cells; LC, Leydig Cells; MC, Mesenchymal Cells.

The scRNA-seq dataset from untreated adult rats was first visualized by t-Stochastic Neighbor Embedding (t-SNE) projection (**Figure 1**). In total, 8 distinguished cell types were identified from 10,648 individual cells of the control rat interstitium (**Figure 1A**). They include (with abbreviations and markers): Leydig cells (LC, *Cyp11a1*), dendritic cells (DC, *Il1r2*), vascular endothelial cells (EC, *Fam110d*), smooth muscle cells (SMC, *Acta2*), mesenchymal cells (MC, *Col1a1*), macrophages (Mac, *Csf1r*), and lymphoid cells (LyC, *Cd3e*). Also, a germ cell cluster (GC, *Insl6*) was detected, which resulted from a contamination in the cell enriching process. In addition to their own specific markers, Mac and DC expressed a common marker, *Cd68*. As expected, all cells expressed ribosomal protein S16 (*Rps16*), a common house-keeping gene, at a similar level.

The identification of the 8 cell types was also confirmed by violin-plots with another set of specific markers (**Figure 1B**). More unique markers can be found from a heatmap plot (**Figure 1C**). The 200 most significant differentially expressed genes (DEGs) for the 7 major cell types were also listed in **Supplementary Tables S2–S8**.

To further analyze the biological significance of the top 200 DEGs, GO and KEGG enrichments were done with the genes for the 6 major cell types (**Figure 1D**, **Supplementary Figure S1**). Some of the top enriched terms for each cell type are highlighted, including LC (xenobiotic/drug metabolism, steroidogenesis, cortisol synthesis, and glutathione metabolism); SMC (cGMP-PKG signaling, smooth muscle contraction, aldosterone synthesis, and various cardiomyopathy); MC (ECM-receptor interaction and cytokine interactions); EC (angiogenesis, cell migration, Notch binding, VEGF response, and apical plasma membrane); Mac (inflammatory response, chemotaxis, immune response, ERK1/2 signaling, and TNF production); and LyC (cytokine interactions, T cell receptor signaling, Th cell differentiation, chemokine signaling, and phagocytosis). These biological processes are consistent with the major biological functions each cell type involves, further confirming the accuracy of the cell identifications. Also, some of these processes have not been recognized previously, such as xenobiotic/drug metabolism by LCs and immune-regulatory roles by MCs.

### Effect of EDS treatment on interstitial cell homeostasis

**Figure 2:**
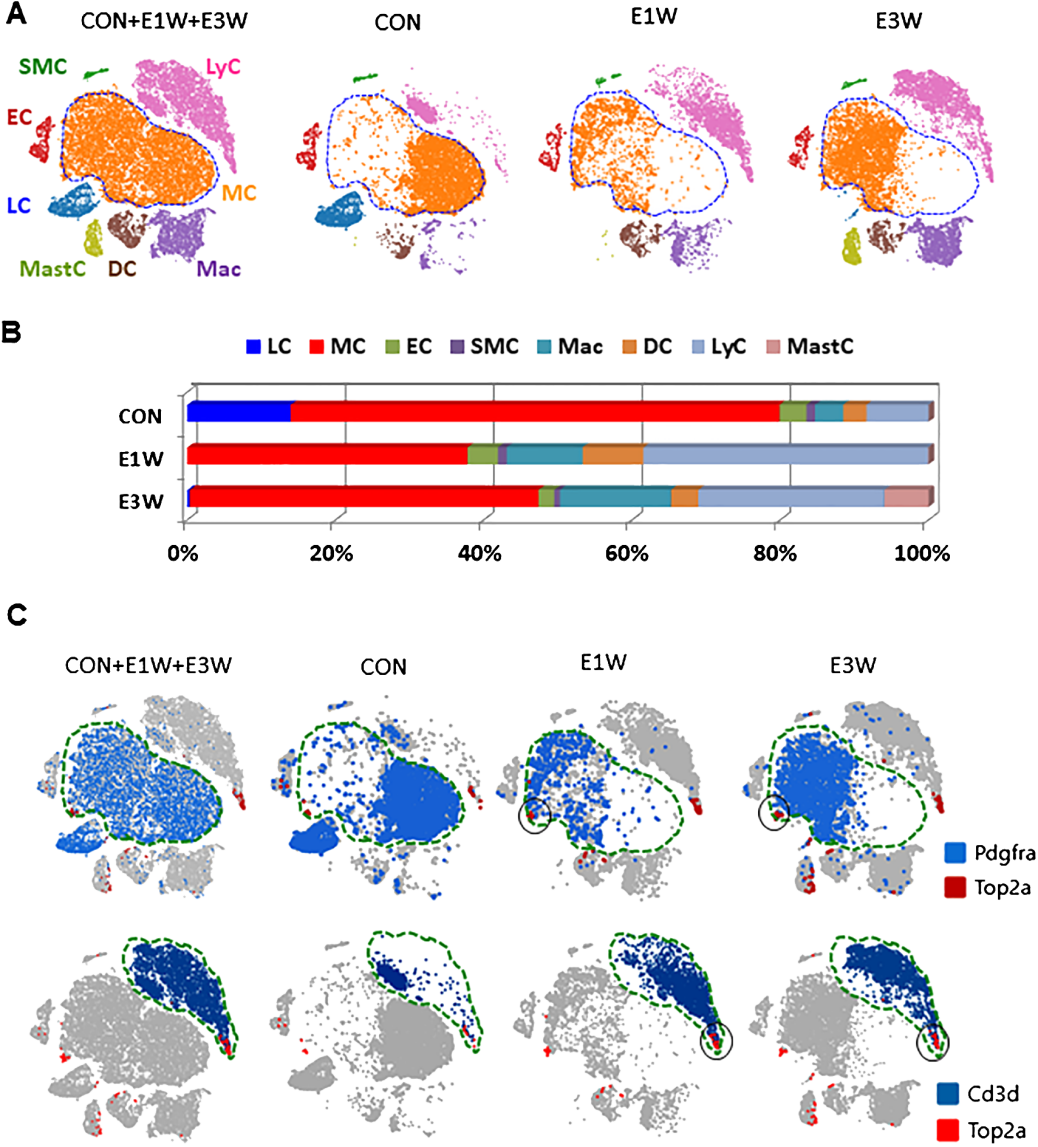
Characterization of interstitial cells of CON and EDS treated (E1W and E3W) samples without including germ cells. **(A)** tSNE plot showing the cell distributions of the three combined samples. Eight unique cell types were identified. **(B)** Cell number differences for each cell type among the three samples. **(C)** Co-expression of division-related gene (*Top2a*) and MC (*Pdgfra*) and LyC (*Cd3d*) markers. The dividing fractions significantly affected by EDS are highlighted by black circles.

With the establishment of cell identities in the control testis (**Figure 1**), we further analyzed the effect of EDS treatment on the interstitial cell populations by comparing samples of control (CON), one (E1W), and three (E3W) weeks post-EDS treatment in a combined dataset (**Figure 2A**). In addition to the disappearance of the LC cluster in the E1W sample, the combined dataset revealed an extra cell type (mast cells) in the E3W, indicating that EDS treatment resulted in an invasion of mast cells by three weeks. Also, cells from the control and EDS samples do not always overlap in the remaining clusters (MC, Mac, DC, and LyC) (**Figure 2A**), suggesting potential changes in transcriptomes and/or cell heterogeneity.

In addition to the loss of the whole LC population and the change of cell distributions for some clusters, EDS treatment also affected the relative numbers of the remaining cells (**Figure 2B**). EDS treatment increased the percentages of the 3 immune cell types (LyC, DC, and Mac) while decreasing the MC population (**Figure 2B**). In addition to affecting cell numbers, EDS treatment also impacted cell activities. The effects of EDS on the transcriptomes of MC, EC, and SMC populations are summarized in **Supplementary Tables S9–S12**. The lists cover partial DEGs for MCs (400/2249) and total DEGs for each of ECs (143/143) and SMCs (29/29). These dramatic differences in the number of total DEGs detected among the 3 cell types suggest strongly that LC elimination by EDS affected MCs more extensively than those of ECs and SMCs.

To further examine the biological connections among the DEGs, GO and KEGG enrichments were carried out with all the DEGs of the 3 cell types (**Supplementary Figures S2-S4**). For MCs as an example, the enriched GO terms for EDS-upregulated genes include extracellular space/matrix, angiogenesis, cell migration, and both positive and negative regulation of cell proliferation (**Supplementary Figure S2C**). The terms associated with the down-regulated genes include extracellular space, GTPase activity, positive regulation of cell proliferation, defense response, cellular response to hormone stimulus, and immune response (**Supplementary Figure S2E**). Overall, EDS increased MC extracellular matrix (ECM) assembly, cell adhesion, and angiogenesis while inhibiting adenosine triphosphate (ATP) synthesis, response to hormonal stimulus, and immune response.

### Effect of EDS treatment on interstitial cell proliferations

It is well-known that EDS treatment triggers interstitial cell proliferation within the first week (29, 30). The cells involved, however, are not well characterized. The cell proliferation was compared between the control and EDS groups using expressions of a group of well-known genes involved in cell proliferation, including *Top2a*, *Mki67*, and *Cenpw* (**Figure 2C**; **Supplementary Figures S5C, D**). A fraction of cells from each cell type, except LC, consistently expressed these 3 genes, indicating their mitogenic states. Cross analysis of *Pdgfra* ^+^ (blue) or *Cd3d* ^+^ cells (blue) with *Top2a* ^+^ cells (red) revealed clear co-localization of the genes in a small fraction of MC and LyC populations (**Figure 2C**, black circles, **Supplementary Figure S5**, red circles). When CON and E1W and E3W samples were compared, the dividing fractions of MCs and LyCs were increased significantly in the EDS groups (**Figure 2C**, black circles).

### Characterization of interstitial populations by protein markers

**Figure 3:**
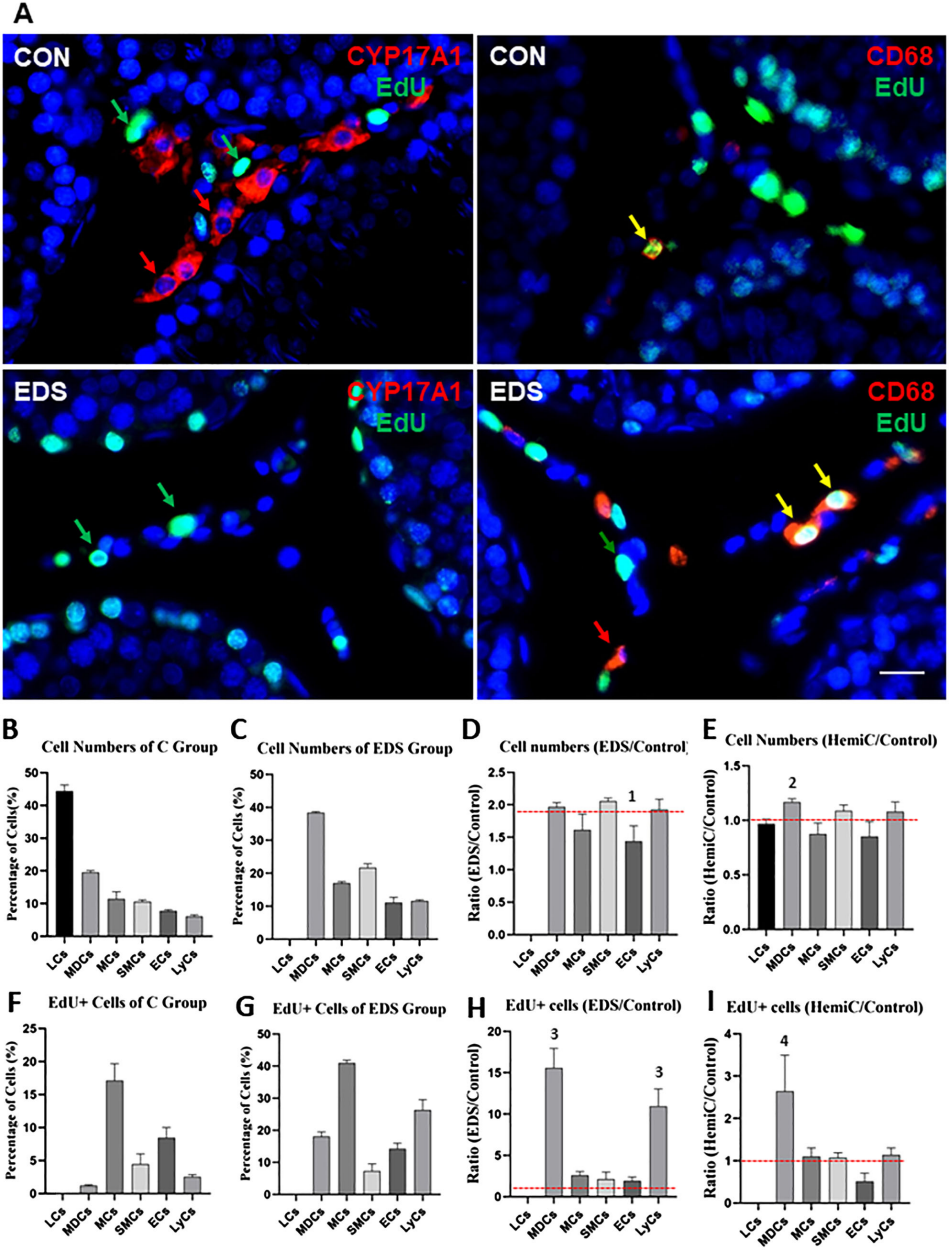
Quantification of six major interstitial cells of control (CON), EDS-treated (E1W) and hemicastrated (Hemi) rats by the specific protein markers. **(A)** Immunofluorescent staining of Leydig Cells (LCs, CYP17A1) or Macrophages and Dendritic Cells (MDCs, CD68) for CON or EDS-treated animals. The detailed staining profiles for other cell types can be found in **Supplementary Figures S6–S8**, including MC: mesenchymal Cells (PDGFRA); SMC: smooth muscle cells (ACTA2); EC: endothelial cells (CD31); LyC: lymphoid cells (CD3). Cells were identified by their marker proteins (red arrow) and the dividing marker (EdU, green arrow). Co-labeled cells are marked by yellow arrows. **(B, C)** Number of each cell type of CON or EDS. **(D, E)** Ratio of the cell number of EDS or hemicastrated rats over the CON rats. **(F, G)** Percentage of the dividing (EdU+) cells of CON or EDS groups. **(H, I)** Ratio of dividing cells of EDS or hemicastrated rats over the control rats. Data are expressed as Mean ± SE of 4 individual animals (n=4). ^1^Significantly different from SMCs at P<0.05. ^2^Significantly different from MCs and ECs at P<0.05. ^3^Significantly different from LCs, MCs, SMCs and ECs at P<0.05. ^4^Significantly different from all other groups at P<0.05. Red broken lines: Neural lines upon which data without change could stay. Scale bars represent 10 μm in length.

Due to technique limitations, cell numbers identified by scRNA-seq procedures may not represent the real numbers *in vivo*. To further characterize the interstitial cell composition of adult rat testes, the major cell types were identified and quantified immunofluorescently with protein markers. The markers used were based on both the results of the current scRNA-seq analyses and previously published studies. The cells analyzed include (with markers): LC (CYP11A1) (22), Mac plus DC (MDC, CD68) (25), LyC (CD3) (26, 27), EC (CD31) (28), SMC (ACTA2) (20), and MC (PDGFRA) (23, 24). The cells were stained red by their specific markers in each case and the dividing nuclei were labeled green (**Figure 3A**; **Supplementary Figures S6–S8**). The numbers of each cell type as a percentage of the total interstitial population are summarized in **Figures 3B–I**. As expected, LC represents the major cell type (44.6%) of the interstitial compartment of control rats, followed by MDC (19.1%), MC (11.5%), SMC (10.7%), EC (7.9%), and LyC (6.2%) (**Figure 3B**). To estimate the dividing rate of each cell type, co-staining of the above markers with EdU were also quantified and expressed as percentages of EdU+ cells within each population (**Figure 3F**). Clearly, MCs have the highest rate of division (17.1%), followed by EC (8.5%) and SMC (4.5%). The immune cells show low proliferation, while LCs are non-proliferative.

### Effects of EDS and hemicastration on interstitial cell homeostasis

To examine how losses of LCs or the contralateral testis may affect the interstitial homeostasis, rats were either treated by EDS or hemicastration. With EDS treatment (loss of LCs), the percentages of all other interstitial cells increased proportionally (**Figure 3D**). Since LCs represent about half (44.6%) of all interstitial cells, elimination of LCs doubled the percentages of all other populations, with the ratio of EDS (**Figure 3C**) over control testes (**Figure 3B**) around 2 (**Figure 3D**). However, the ratios for ECs were significantly lower than one or more of the four other cell types, suggesting a specific effect of EDS on this particular population. Also, as expected, EDS treatment not only affected the cell numbers but also increased their proliferations (**Figure 3F** vs **3G**). The cell type with the highest dividing activity in the EDS animals is still MC (about 41%, **Figure 3G**). However, the ratios (**Figure 3H**) of the EDS and control testes indicated a surprising finding: EDS treatment increased the division of immune populations (MDC and LyC, more than 10-fold increases) far greater than other somatic cells (2-3 fold). In contrast to EDS treatment, hemicastration did not affect either cell numbers or cell division dramatically, except for mild increases in MDC numbers (**Figure 3E**; **Supplementary Figure S8**) and proliferation (**Figure 3I**; **Supplementary Figure S8**).

As previously reported, serum testosterone concentration was maintained at a level comparable to the control rats despite the castrated rats having only one testis (**Supplementary Figure S9A**). To examine whether hemicastration affected steroidogenic enzyme or immune cells, we compared the marker protein expressions of the 3 major cell types (LC, MDC, and LyC) by western blots (**Supplementary Figure S9B**). Consistent with the immunofluorescent results, the expression of the Leydig cell marker protein CYP17A1 was lost completely one week after EDS treatment but recovered fully by 8 weeks (EDS 8w). However, 2 immune cell marker proteins, CD68 and CD3, were not significantly affected by EDS treatment. Also, hemicastration did not affect the expressions of any of the 3 markers. Overall, these results support the immunofluorescent observations, indicating that the interstitial cell homeostasis is significantly affected by EDS treatment but not by short-term hemicastration.

### Leydig cell turnover and replacement in adult rat testis

Based on the findings that LCs of adult testis do not divide, we wanted to further examine whether the cells die under normal circumstances. Co-staining of cells with CYP17A1 and apoptotic nuclei by TUNEL staining revealed double positive cells (**Supplementary Figure S9C**, white arrow), suggesting that although adult LCs do not divide, they do die. Co-staining of CYP17A1 and EdU for 8-week EDS samples (EdU-labeling was done during the first week after EDS treatment) detected double positive cells (**Supplementary Figures S9D, E**), confirming that the dividing cells right after EDS treatment eventually formed LCs.

### Characterization of MC heterogeneity and effects of EDS treatment

**Figure 4:**
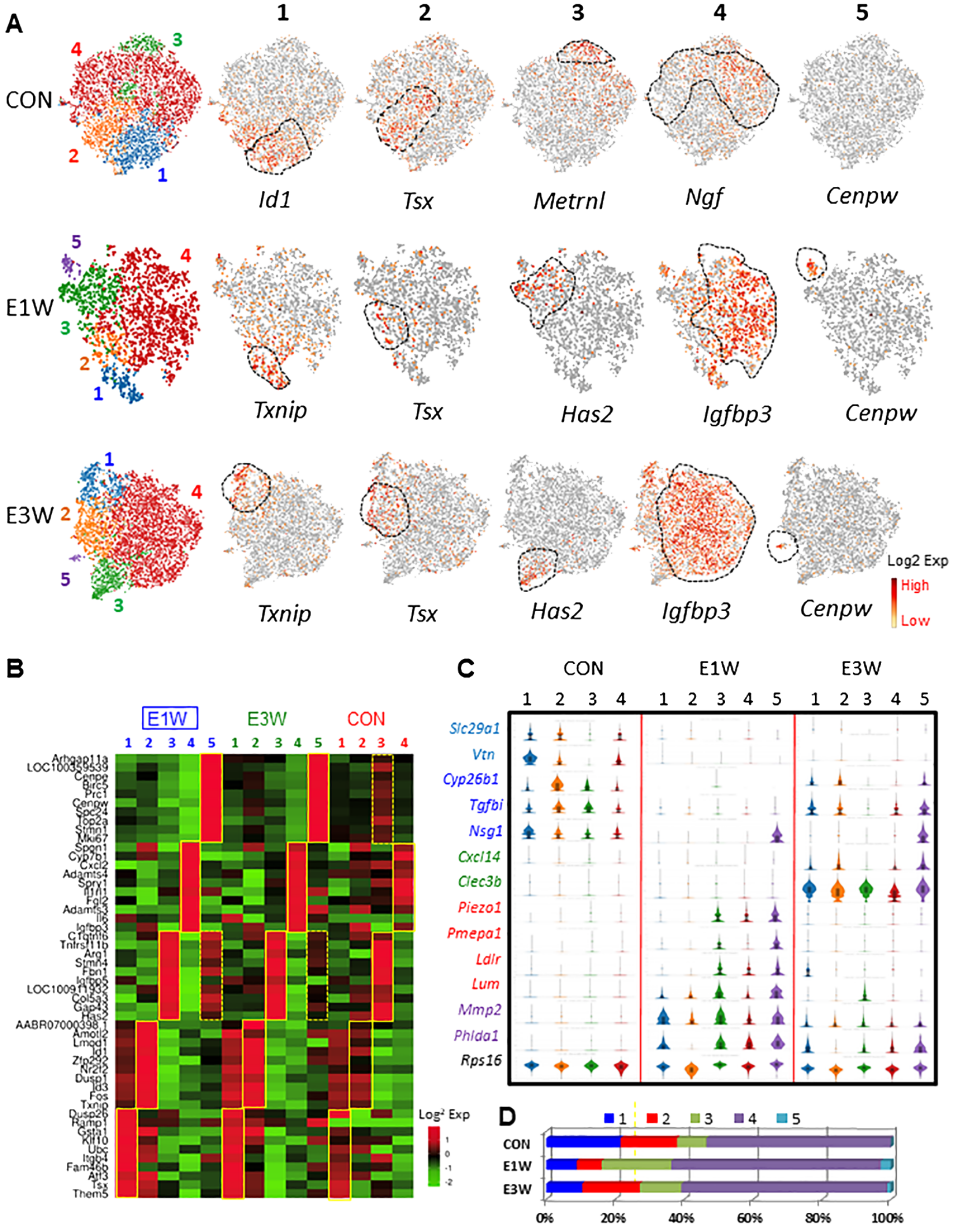
Characterization of MC subgroups and effects of EDS on the cells. **(A)** MCs were further clustered into 4 (control, CON) or 5 (E1W and E3W) subgroups. Each cluster has enriched genes. **(B)** Expression of the top 10 DEGs of 5 subgroups of E1W (E1-E5) by CON and E3W groups. **(C)** Violin distributions for some of the genes significantly affected by EDS treatment. **(D)** Relative cell numbers of the 4 or 5 subpopulations between CON and EDS samples.

To characterize the heterogeneity of mesenchymal cells, we have re-clustered the population further for each of the control, E1W, and E3W groups (**Figure 4A**). Four and five sub-clusters were generated for the control (CON) and EDS groups, respectively. The representative genes enriched for the subclusters of the CON and EDS groups were compared, including *Id1* (C1), *Tsx* (C2), *Metrnl* (C3), *Ngf* (C4), *Txnip* (E1), *Tsx* (E2), *Has2* (E3), *Igfbp3* (E4), and *Cenpw* (E5). To study the difference and similarity of the clusters identified for both the CON and EDS groups, the top 10 DEGs for each cluster were compared across the 3 groups (CON, E1W, and E3W) (**Figure 4B**; **Supplementary Figure S10**). For the 40 DEGs of the CON groups (C1-C4), majority were also enriched in each of the 4 corresponding clusters in the EDS groups (CON vs E1W or E3W) except those in cluster 1 and 2 (C1 and C2) lost their specificities between the E1 and E2 clusters of the E1W group, suggesting that EDS treatment somehow diminished the difference between the C1 and C2 clusters (**Supplementary Figure S10**).

When the DEGs of the 5 EDS clusters (E1-E5) were compared back to the 4 control clusters (C1-C4) (**Figure 4B**; **Supplementary Figure S10**, E1W or E3W vs CON), similar enrichments were also found for the corresponding clusters of the CON groups, but with less robust enrichments compared to (not as red as) E1W vs EDS (**Figure 4B**). Also, the DEGs of the newly appeared E5 cluster (dividing cells) were enriched specifically by cluster 3 of the control (C3, boxes with broken line), suggesting that the dividing cells of the EDS groups were most likely derived from the C3 cluster of the CON group.

Some of the DEGs among the three groups were also compared by their violin-distributions (**Figure 4C**). Genes expressed highly in the CON but lowly in one or two EDS groups are shown in light or bright blue colors. Genes uniquely expressed in the E3W group are shown in green. Genes with high expressions in the E1W are shown in red. Genes with high expressions in both EDS groups are shown in purple. *Rps16* was evenly expressed by all 14 clusters across the three groups.

In addition to changes in the transcriptome of each cluster, EDS also affected the cell number of the individual cluster (**Figure 4D**). In the CON, the cluster 4 represents 53.5% of the whole population, followed by cluster 2 (16.4%), 1 (21.6%), and 3 (8.5%). One week after EDS treatment, clusters 1 and 2 decreased significantly while cluster 3 increased (almost tripled). Also, cluster 5 containing dividing cells (3.0%) appeared. By 3 weeks after EDS treatment, cluster 2 recovered while cluster 1 did not. Also, cluster 3 reduced in size compared to E1W but was still larger than the one in the CON. The dividing cluster reduced in size. These results suggest clearly that EDS affected MC heterogeneity.

### Characterization of biological functions associated with the MC subgroups

**Figure 5:**
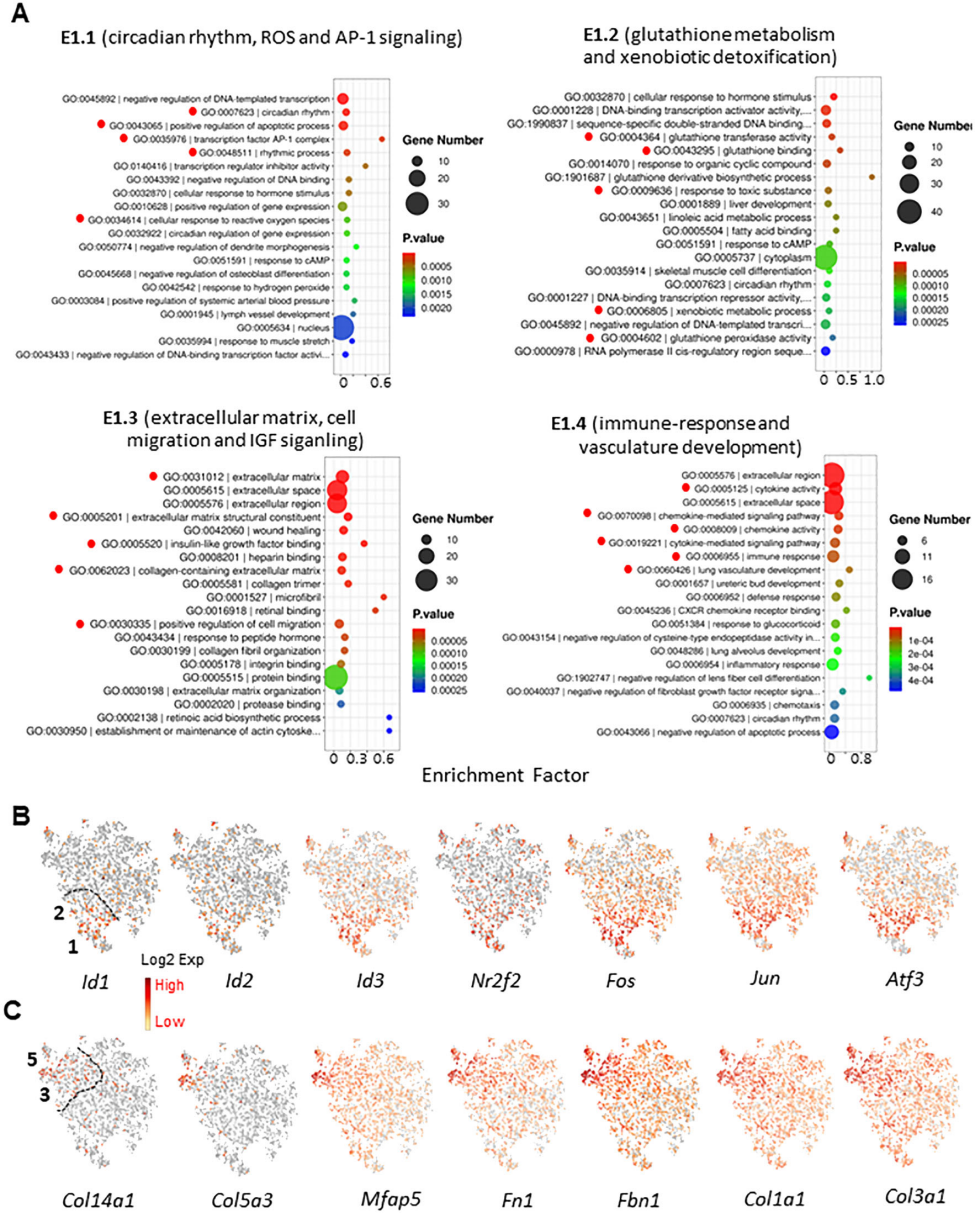
Functional enrichment of MC subgroups. **(A)** Top 20 GO terms enriched of the top 100 DEGs for the 4 MC subgroups of E1W sample. Important terms were high-lighted by red dots. **(B)** Enrichment of nuclear protein genes for subgroups 1 and 2. **(C)** Enrichment of ECM-related genes for subgroups 3 and 5.

To further characterize the unique functions of the MC subclusters, we have done GO and KEGG enrichments with the top 100 DEGs identified for each cluster (**Figure 5A**; **Supplementary Figures S11-S13**). The enrichments revealed clear distinguished features for most clusters. For the 5 EDS clusters (E1W) as an example, the major features enriched for the 5 clusters were: E1, circadian rhythm, antioxidants, and AP-1 signaling; E2, glutathione metabolism and xenobiotic detoxification; E3, ECM synthesis and cell migration; E4, immune-response and regulation; E5, cell cycle and cell division. These results suggest that testicular MCs have a high degree of heterogeneity with specialized functions by different subgroups.

In addition to the major functions listed above, we have noticed that DNA binding and transcriptional stimulation and inhibition were also enriched by E1 and E2. Further analysis revealed that three forms of inhibitor of DNA binding gene (*Id1-3*) and AP-1 subunits (*Fos* and *Jun*) were enriched by the two clusters (**Figure 5B**). This unique expression is also related to the enrichment of circadian rhythm by the two clusters (**Figure 5A**). In contrast to the unique transcriptional factors enriched by E1 and E2, multiple ECM genes were enriched by clusters 3 and 5 (E3 and E5) (**Figure 5C**). The unique expression of these genes is consistent with the GO enrichment of ECM-associated biological functions and cell migration by the cluster 3 (**Figure 5A**).

**Figure 6:**
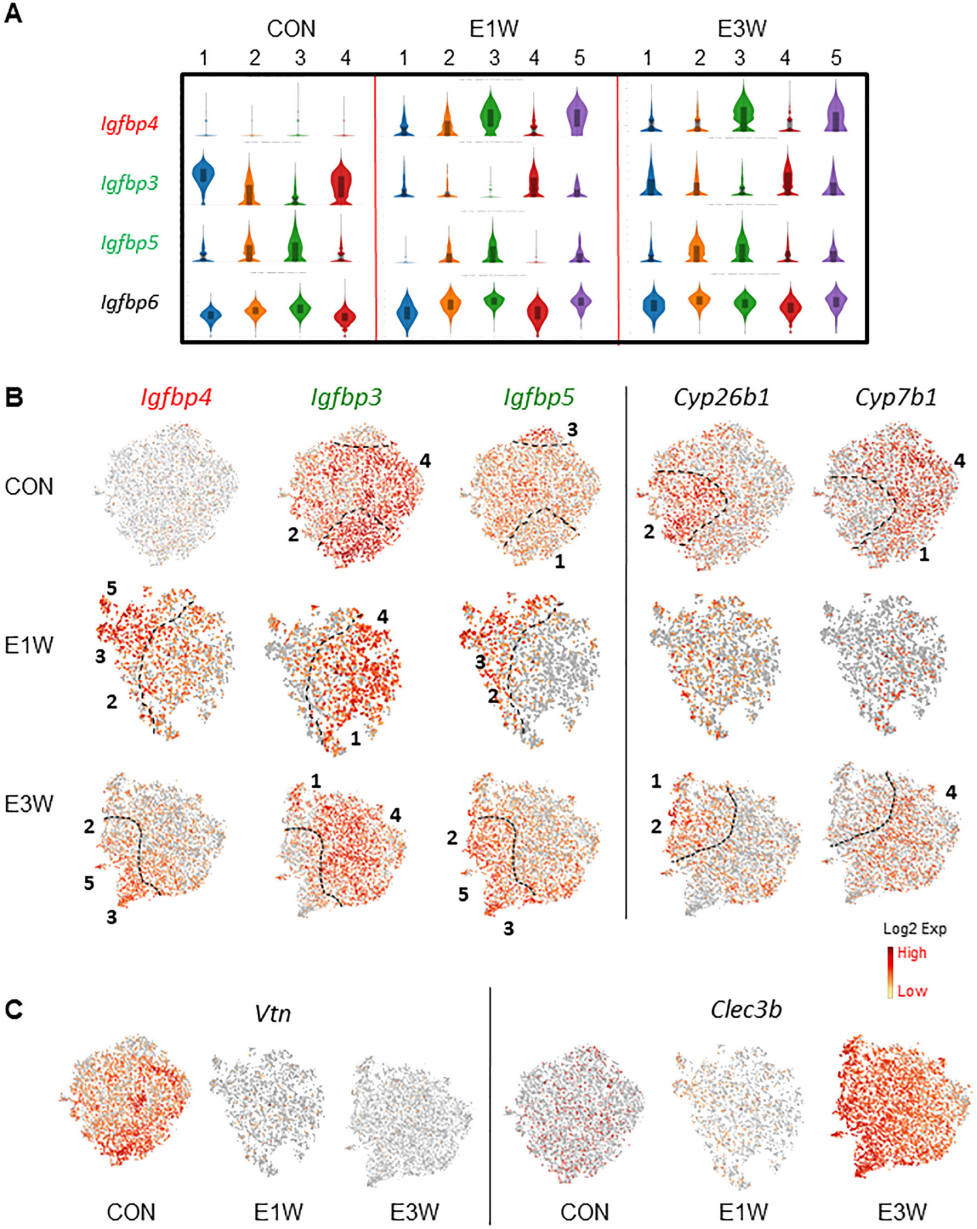
Effect of EDS (E1W or E3W) on the expression of *Igfbp* and detoxification genes in MC subpopulations. **(A)** Expression of 4 *Igfbp* genes, with EDS-related DEGs being highlighted by red (increased), green (reduced) or black (unchanged) boxes. **(B)** tSNE plots of the 3 *Igfbp* genes and 2 detoxification genes with mutual exclusive expressions. **(C)** tSNE plots of genes turned off (*Vtn*) or on (*Clec3b*) by EDS treatments.

Another group of genes with clear distinguished expression patterns are insulin-like growth factor binding proteins (*Igfbp*) (**Figures 6A–C**). Among the 6 members of the family that were detected in MCs (*Igfbp2-7*), *Igfbp4* was increased while *Igfbp3* and *Igfbp5* were decreased by EDS treatment (**Figure 6A**). Further comparison of the three genes found that *Igfbp3* and *Igfbp4* were affected universally across all 4 clusters by EDS treatment, while *Igfbp5* was reduced specifically in cluster 2 and 4 of the E1W group (**Figures 6A, B**). Interestingly, the complementary expressions of *Igfbp3* and *Igfbp5* were further enhanced by EDS treatment, so the expression of the two genes became completely mutually exclusive in different subgroups of the EDS samples (**Figure 6B**), indicating that LC loss significantly affected the unique expressions of *Igfbp3* and *Igfbp5*.

The mutually expressing pattern was also found for genes in the CON cells (**Figure 6B**). For example, *Cyp26b1* and *Cyp7b1* form a mutually exclusive expression pattern, with *Cyp26b1* primarily expressed by cluster 2 and *Cyp7b1* expressed by clusters 1 and 4. Although both enzymes play roles in xenobiotic detoxification, CYP26B1 can metabolite all-trans retinoic acid (RA) while CYP7B1 cannot, suggesting that RA signaling may be selectively regulated in certain subpopulations by upregulation of CYP26B1. Also, this unique expression pattern was reduced one week after EDS treatment but returned by 3 weeks after EDS, suggesting that loss of Leydig cells may affect the xenobiotic detoxification capacity and/or RA signaling of the mesenchymal population in the short term. Also, in addition to the genes up-regulated by EDS treatment (*Igfbp4* and *Clec3b*) (**Figure 6A–C**), there were also genes that were completely inhibited by EDS, such as *Vtn* (**Figure 6C**).

### Identification of potential SLC-associated subgroups and effects by EDS treatment

**Figure 7:**
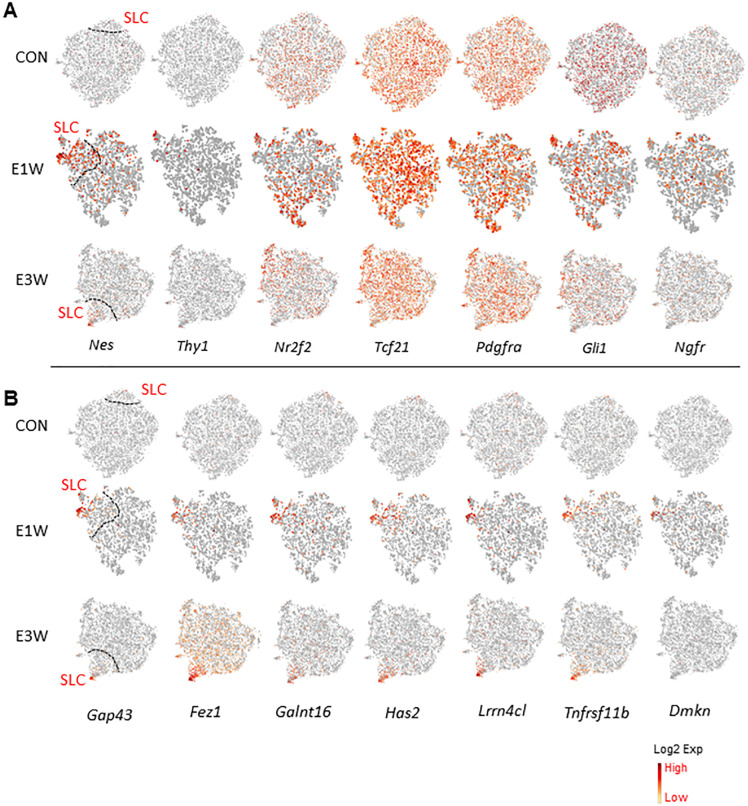
Expression of SLC-related genes by MC subgroups. **(A)** SLC marker genes reported previously. **(B)** Genes enriched by SLC-associated subgroup of EDS animals.

It is well known that the MC population contains SLCs, which are responsible for LC generation during puberty and adulthood. To examine which of the four subpopulations may contain SLCs, we applied the seven SLC marker genes reported previously to the four mesenchymal populations (**Figure 7A**). In the CON group, except for *Nes* and *Thy1*, all other genes were detectable. However, they were evenly distributed across the four clusters. In the EDS groups, all genes were detected, including *Nes* and *Thy1*. Among the seven genes, only *Nes* and *Thy1* were specifically expressed by cluster 3 and partially by cluster 5. Since EDS treatment triggers the proliferation of SLCs, cluster 5 (dividing cells) most likely represents the true SLCs. Also, since E5 is transcriptomically closer to E3 than to all other clusters (**Figure 4B**; **Supplementary Figure S10**), it is reasonable to assume that E3 and E5 are the major SLC-containing clusters.

To identify new and more specific SLC marker genes, we explored all the DEGs associated with clusters 3 and 5. Some of the genes enriched in cluster 3 of the CON group are shown in **Supplementary Figure S14**, and those for the EDS groups are shown in **Figure 7B**. Interestingly, these genes showed specific expressions in the CON group, and their expressions increased and became more diffuse after EDS treatment, while those with specific expressions in the EDS groups were almost undetectable in the CON group, suggesting that EDS treatment somehow up-regulated the SLC featured genes.

**Figure 8:**
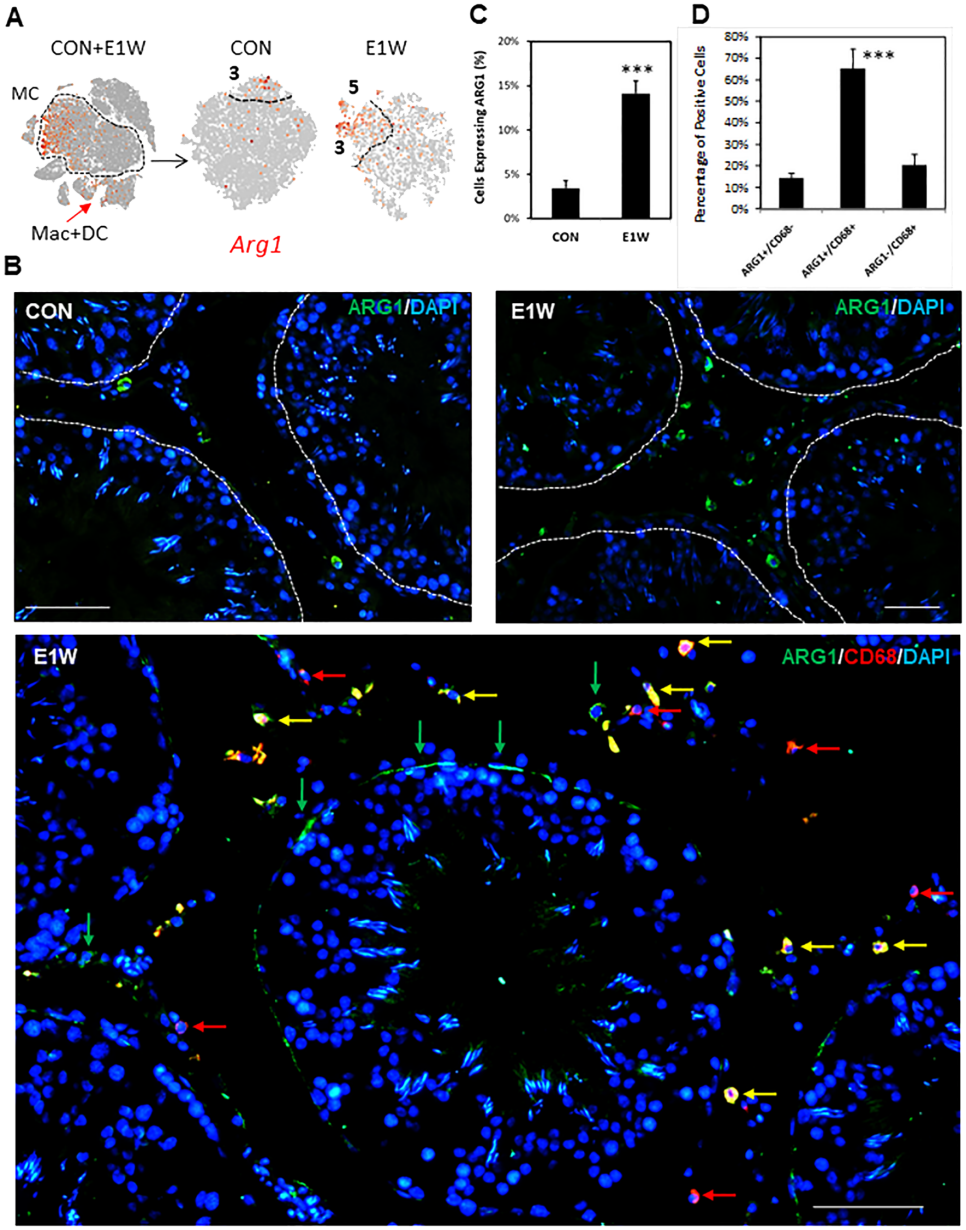
Immunofluorescent detection of ARG1^+^ cells in CON and EDS animals. **(A)** Cluster-dependent expressions of the *Arg1* gene by MCs of the CON and E1W groups. **(B)** Immunofluorescent detection of ARG1^+^ cells in the CON and E1W groups, and the co-staining of ARG1 and CD68. Green arrows: ARG1^+^/CD68^-^ cells; Red arrows: ARG1^-^/CD68^+^ cells; Yellow arrows: ARG1^+^/CD68^+^ cells. **(C)** Percentage of ARG1^+^ cells in the entire interstitial population in the CON and E1W groups. **(D)** Percentage of ARG1^+^ and/or CD68^+^ cells. Data are expressed as Mean ± SE of 3 individual animals (n = 3). ***Significantly different from CON at P < 0.001. Scale bars represent 50 μm in length. (A larger view of the staining can be found in **Supplementary Figure S15**).

**Figure 9:**
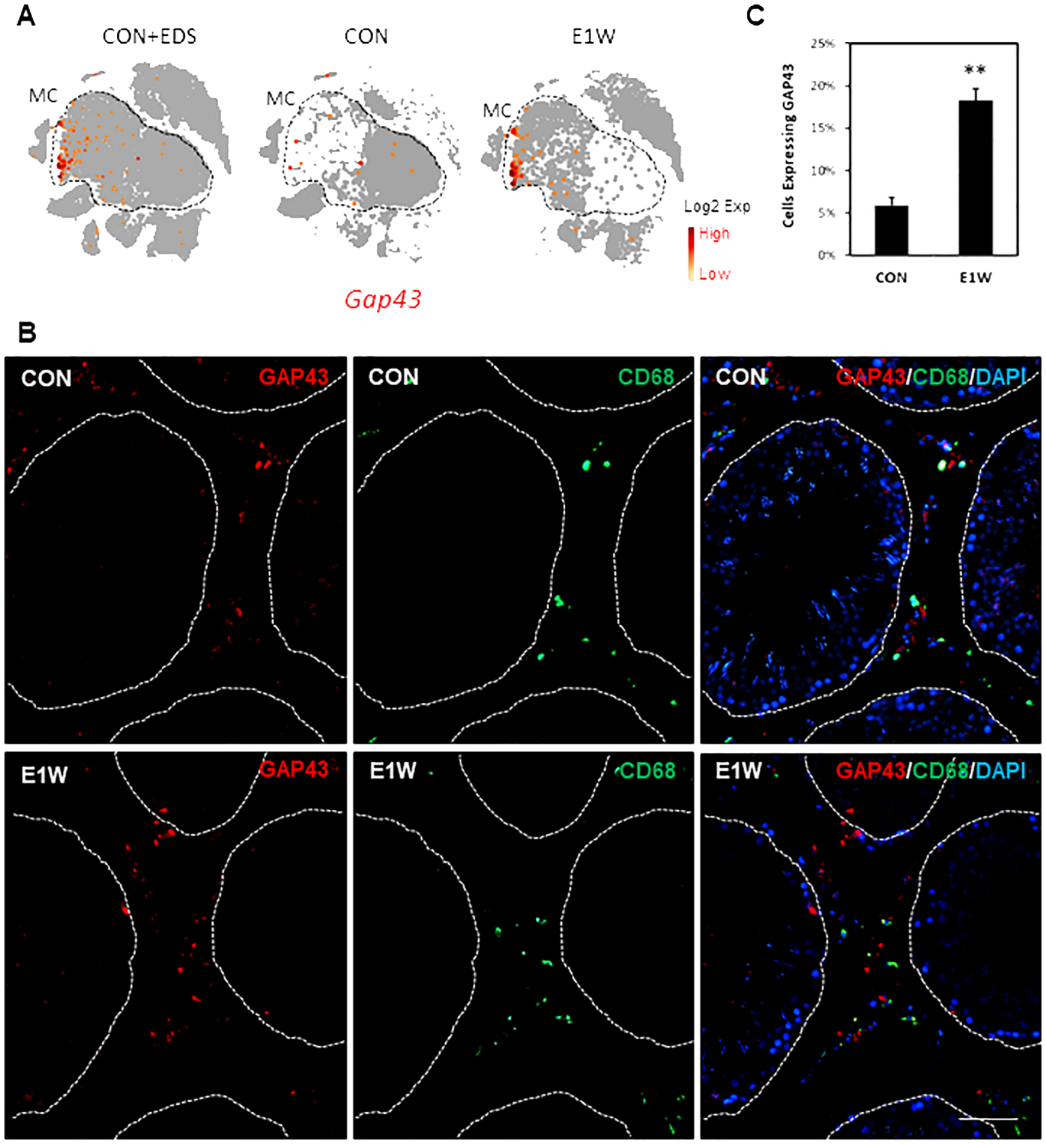
Immunofluorescent staining of GAP43^+^ cells in CON and EDS animals. **(A)** Cluster-dependent expressions of the *Gap43* gene by MCs of the CON and E1W groups. **(B)** Co-staining of GAP43 and CD68 of the CON and E1W animals. Red: GAP43^+^ cells; Green: CD68^+^ cells. No co-staining was detected. **(C)** Percentage of GAP43^+^ cells in the entire interstitial population in the CON and E1W animals. Data are expressed as Mean ± SE of 3 individual animals (n = 3). **Significantly different from CON at P < 0.01. Scale bars represent 50 μm in length. (A larger view of the staining can be found in **Supplementary Figure S17**).

To confirm some of the SLC-enriched genes at the protein level, we did immunofluorescent staining of ARG1 (**Figure 8**; **Supplementary Figure S15**) and GAP43 (**Figure 9**; **Supplementary Figure S17**), whose genes are highly expressed in cluster 3 and/or 5. FN1, whose gene is universally expressed in the MC population, was also examined (**Supplementary Figure S18**). At the RNA level, *Arg1*-expressing MCs are scarce and enriched in cluster 3 of CON and E1W groups (**Figure 8A**). The scarcity is confirmed by the protein staining of CON and E1W testes (**Figure 8B**; **Supplementary Figure S15**). Quantification shows that about 3.5% of interstitial cells were ARG1-positive in CON, and it increased to 14.1% in E1W (**Figure 8C**). Since *Arg1* is also weakly expressed by macrophages and dendritic cells (**Figure 8A**, arrow), we did co-staining of ARG1 and CD68 (**Figure 8B**, bottom). Surprisingly, most ARG1^+^ cells were CD68-positive. Quantification from three E1W animals indicates that double positive cells were about 65%, while Arg1^+^/CD68^-^ and ARG1^-^/CD68^+^ cells were about 15% and 20% respectively (**Figure 8D**).

Regard GAP43 (**Figure 9**; **Supplementary Figure S17**), the percentage of positive cells was around 6% in CON and tripled to about 18% in E1W animals (**Figure 9C**). Co-staining with the CD68 antibody detected no co-localization, supporting the marker’s specificity for potential SLCs. FN1 staining showed a broad protein expression across the interstitial compartment, including peritubular cells (**Supplementary Figure S18**), which may be related to extracellular deposition of the protein as FN is an important ECM component.

## Discussion

Rodents have been widely used by researchers over the years to understand mammalian reproductive biology. In contrast to cells associated with the seminiferous tubular compartment, the testicular interstitial cells are not well-defined. In addition to the well-established cells, such as Leydig cells and peritubular and interstitial macrophages (31–34), more immune cells have been identified in the interstitial compartment (35). However, their exact numbers and turnover dynamics remain unknown. In the current study, we quantified the number of 7 major interstitial cell types and their dividing activities by continuous EdU exposure for a week.

As expected, Leydig cells (LCs) represent the major cell type (44.6%) of the interstitial compartment, followed by macrophages and dendritic cells (MDCs, 19.1%), mesenchymal cells (MCs, 11.5%), smooth muscle cells (SMCs, 10.7%), vascular endothelial cells (ECs, 7.9%) and lymphoid cells (LyCs, 6.2%). The EdU label experiment indicated that MCs have the highest rate of division (17.1%), followed by ECs (8.5%) and SMCs (4.5%). The immune cells showed low proliferations (LyCs, 2.5%; MDCs, 1.2%), while LCs are non-proliferative.

### Effects of EDS treatment on interstitial cells homeostasis

With LC elimination by EDS treatment, the other 6 cell types were still visible in the scRNA-seq dataset, suggesting that except for the disappearance of LCs, EDS treatment did not eliminate other cell types. However, EDS treatment resulted in the invasion of mast cells by 3 weeks. This observation is consistent with a previous finding (36). Also, EDS treatment affected the cell distributions of certain clusters (MC, Mac, DC, and LyC) more than others (EC or SMC), suggesting profound alterations in cell transcriptomes and/or heterogeneity of the former. Indeed, EDS treatment induced multiple changes in the transcriptome for each of the 3 major interstitial cell types, MC, SMC, and EC. However, the number of differentially expressed genes (DEGs) detected for MC (2241) is far greater than those of SMC (29) or EC (143), indicating that EDS may affect MC far more severely than the other two cell types (the effects on MC homeostasis will be discussed in more depth below).

### Effects of EDS treatment on immune cells

Immune cells are among the interstitial populations affected the most by EDS treatment. It is known that Leydig cell elimination and the regeneration after EDS treatment rely on macrophages (34, 37). However, the effects of EDS treatment on other immune cells are less well understood. In the current study, EDS treatment activated the immune responses of ECs in such a dramatic way that among the top 20 GO processes enriched, 12 were related to immune/inflammation (**Supplementary Figure S4A**). This finding suggests strongly that ECs may be among the most sensitive immune-responding cell types in the interstitial compartment induced by EDS treatment.

However, it is a little surprising to find that EDS treatment did not affect the immune cell numbers dramatically by one week. Since the immune cell proliferations were up-regulated dramatically at that time, the number of immune cells could change in the longer term. This is consistent with previous observations that immune cell composition changed, including mast cell invasion, in 3-7 weeks after EDS treatment (38). Also, these long-term changes in immune cells most likely resulted from LC loss, not from EDS treatment itself, since inhibition of LC recovery also prevented normalization of immune cells in the later stage (38).

At least two major adult testicular macrophage populations have been reported: CSF1R^+^CD206^+^MHCII^-^ macrophages that are present in the interstitial parenchyma; and CSF1R^-^CD206^-^MHCII^+^ macrophages that are localized to the surface of seminiferous tubules (31, 32). Unfortunately, the resolution of the current datasets did not go deep enough to cover all these key genes, so the character of the two macrophages in the current study is still unclear. Since the interstitial cells were pre-enriched to eliminate tubular cells, we do not expect the presence of seminiferous tubular macrophages in the present study. However, even with the pure interstitial cells, we have noticed evidence of macrophage heterogeneity. For example, about 80% of *Adgre1* ^+^ macrophages expressed a high level of *Pdgfc*, while another 20% did not express *Pdgfc* at all.

It is well-known that testosterone can modify testicular immune cell functions intensively. For example, testosterone stimulates the differentiation of primitive T cells into immunosuppressive Tregs and prevents the infiltration of inflammatory macrophages (39). Testosterone also stimulates the phenotypic transformation of M1 macrophages to M2 macrophages induced by CSF2 (40), and maintains the M2 phenotype of macrophages (41). Elimination of LCs by EDS treatment could be expected to affect macrophage phenotypes. The potential effect of changes in the testicular interstitial micro-environment (including EDS treatment) on testicular immune cells is currently under study in our lab.

### Mesenchymal cell heterogeneity and its response to Leydig cell elimination

Since the MC population is another cell type mostly affected by EDS treatment, we have analyzed the population in depth. Unsupervised clustering of the MC population resulted in 4 subgroups with distinguished marker genes and biological functions for both control and EDS-treated groups, except that the latter had an extra cluster containing dividing cells. EDS treatment had profound effects on clusters 1 and 2, resulting in significant loss of cells in the two clusters and the top 10 markers distinguishing the two. Since the major functions of the two clusters involve stress-response and detoxification, the current results indicate that EDS treatment profoundly affected MC sub-fractions responsible for relieving stress and detoxification.

In addition to specific changes targeting certain clusters, EDS treatment also affected the whole MC population. Other strikingly differences between the control and EDS samples involved individual genes whose expressions were either completely inhibited or newly induced. For example, the expression of *Vtn* was completely shut off by EDS treatment, while *Igfbp4* and *Clec3b* were induced in cells crossing the whole MC population. VTN is a multifunctional extracellular matrix (ECM)-related protein that plays significant roles in cell adhesion, migration, survival, and differentiation (42). Also, VTN can cross-link pathogens and integrin receptor and therefore plays a crucial role in innate immunity (43). In addition, VTN can function as a critical complement regulatory protein to prevent hazardous complement activation toward host tissue. IGFBP4 is one of the seven IGF-binding proteins involved in IGF-related metabolism and growth. Induction of this protein suggests strongly that the loss of LCs affected the IGF signaling in MCs. The significance of these striking changes in individual genes deserves further study.

### Is there a specific MC subpopulation associated with stem Leydig cells?

It is well known that elimination of adult Leydig cells (ALCs) by EDS increased stem Leydig cell (SLC) proliferation within the first week of the treatment (29, 30), and the dividing MCs (cluster 5) might represent true SLCs. Transcriptomically, cluster 5 is more closely associated with cluster 3, which was also enriched with the two well-known SLC markers *Nes* and *Thy1* (16, 17, 19). Other markers were either undetected or not enriched by any particular cluster. The conclusion of cluster 3 as true SLCs is also supported by two other observations. First, GO and KEGG enrichments suggest that unlike other clusters, cluster 3 was not associated with any specific biological functions except ECM synthesis and IGF signaling. It is well-known that *in vivo*, one of the most significant biological changes during the early stage of Leydig cell development is about ECM-related cell adhesion and migration (44). Second, the enrichment of IGF-signaling by the cluster is also consistent with the early observations that IGF-1 and IGF1R are essential in the division and differentiation of SLCs and progenitor LCs (45, 46). These evidences all support the conclusion that the cluster 3 contains SLCs.

To confirm the enriched expressions of cluster 3-associated genes, we examined the protein expressions of ARG1, GAP43, and FN1 by immunofluorescent staining of testis sections. Results indicated that both ARG1 and GAP43 were expressed by relatively rare interstitial cells, which were significantly increased by EDS treatment. However, unlike GAP43, the majority of ARG1^+^ cells also expressed CD68, suggesting that most of these cells may actually be macrophages and/or dendritic cells. This is consistent with a previous finding that ARG1 was expressed by macrophages (47). However, there were still about 15% ARG1^+^ cells that were negative for CD68, especially for those located peritubularly, suggesting that this population may represent true SLCs. In contrast, FN1 is universally expressed by all MCs and other cell types, such as peritubular cells. These results confirmed the differential expressions of cluster 3 genes by protein markers. Further studies of their LC differentiation potentials are required to firmly establish their characters as SLC markers.

### Effects of hemicastration on interstitial cells homeostasis

In contrast to the dramatic changes in both cell numbers and proliferative activity following EDS treatment, hemicastration did little to the interstitial homeostasis of the remaining testis except for mild increases in the number and dividing activity of macrophages and/or dendritic cells within a week. These changes suggest that although hemicastration did not affect serum testosterone, it may still affect the immune cells of the testis. The observation that serum testosterone was maintained at a physiologically normal level despite only one functional testis is consistent with previous findings (48–52). Instead, the Leydig cells increased in volume and steroidogenic capacity in response to hemicastration (51, 53). New techniques, such as scRNA-seq and other “Omics” approaches, may be required to explore the molecular mechanisms involved in hemicastration.

In summary, the adult rat testicular interstitial compartment contains seven cell types, including Leydig, mesenchymal, vascular endothelial, smooth muscle, dendritic and lymphoid cells, and macrophages. The overall turnover rate of the interstitial cells is low except for the mesenchymal cells. Also, the mesenchymal cell type is a highly heterogeneous population containing at least four subgroups with distinguished functions. Stem LCs are associated with subgroup 3, which is primarily involved in ECM synthesis, cell binding and migration, and is regulated by the IGF family. Elimination of Leydig cells affected the mesenchymal and immune cells the most, suggesting that Leydig cells may play roles in maintaining the homeostasis of other interstitial populations. Future works are needed to elucidate the diversified functions each interstitial cell may have and how Leydig cells regulate the overall homeostasis of the interstitium.

## Data Availability

The scRNA-seq raw data in fastq (.fq) format can be obtained from the NDGC/GSA repository (https://ngdc.cncb.ac.cn/gsa/s/2QvgQU09). The processed expression matrices (*.mtx) files and the DEG lists can be accessed from the NDGC/OMIX repository (http://ngdc.cncb.ac.cn/omix/preview/yRPuVbr5). The original data for other parts are available from the corresponding author upon request.
