# Supplementary material for: Effects of Leydig cell elimination on testicular interstitial cell populations: characterization by scRNA-seq and immunocytochemical techniques
Source: Front Endocrinol (Lausanne). 2024 Aug 20;15:1423801. doi: 10.3389/fendo.2024.1423801 (PMC11368788; doi:10.3389/fendo.2024.1423801)
Supplement: Supplementary file 1 [file DataSheet1.docx]

Supplemental Materials for Huang et al., Effects of Leydig Cell Elimination on Testicular Interstitial Cell Populations: Characterization by scRNA-seq and Immunocytochemical Techniques

There are 21 supplemental tables and 17 supplemental figures included.

**Table S1. Manufacture and Dilution of Antibodies**

| **Antigen** | **Antibody** | **Dilution** | **Manufacturer (Cat#)** |
| --- | --- | --- | --- |
| Anti-Rabbit | Goat IgG (H+L) HRP | 1:2000 | MULTI SCIENCES (GAR007) |
| Anti-Mouse | Goat IgG (H+L) HRP | 1:2000 | MULTI SCIENCES (GAM007) |
| Anti-Rabbit | Goat Dylight 488 | 1:500 | MULTI SCIENCES (SA00013-2) |
| Anti-Mouse | Goat Dylight 488 | 1:500 | MULTI SCIENCES (SA00013-1） |
| Anti-Rabbit | Goat Dylight 594 | 1:500 | MULTI SCIENCES (SA00013-4） |
| Anti-Mouse | Goat Dylight 594 | 1:500 | MULTI SCIENCES (SA00013-3） |
| β–Actin | Mouse monoclonal | 1:1000 | Beyotime Biotech (AA128-1) |
| CYP11A1 | Rabbit mAb (IF) | 1:500 | Cell Signaling Technology (#14217) |
| CYP17A1 | Rabbit monoclonal(WB) | 1:1000 | Cell Signaling Technology (#94004) |
| CYP17A1 | Rabbit monoclonal(IF) | 1:500 | Cell Signaling Technology (#94004) |
| CD31 | Mouse monoclonal (IF) | 1:500 | Santa Cruz Biotechnology (sc-376764) |
| CD68 | Mouse monoclonal (IF) | 1:500 | Santa Cruz Biotechnology (sc-20060) |
| CD68 | Mouse monoclonal (WB) | 1:1000 | Santa Cruz Biotechnology (sc-20060) |
| CD3 | Rabbit monoclonal(IF) | 1:500 | Abcam (ab16669) |
| CD3 | Rabbit monoclonal (WB) | 1:1000 | Abcam (ab16669) |
| PDGFRA | Rabbit monoclonal (IF) | 1:500 | Abcam (ab203491) |

ACTA2 Rabbit polyclonal (WB) 1:1000 Abcam (ab5694)

Arg1 Goat polyclonal (IF) 1:500 Novus (NB100-59740)

FN1 Sheep polyclonal (IF) 1:500 R&B (AF1918)

Gap43 Sheep polyclonal (IF) 1:500 Novus (NBP1-41123)

**Table S2: Top 200 significantly up-regulated genes of Leydig cells in comparison to all other cell types**

**Table S3: Top 200 significantly up-regulated genes of mesenchymal cells in comparison to all other cell types**

**Table S4: Top 200 significantly up-regulated genes of endothelial cells in comparison to all other cell types**

**Table S5: Top 200 significantly up-regulated genes of smooth muscle cells in comparison to all other cell types**

**Table S6: Top 200 significantly up-regulated genes of macrophages in comparison to all** **other cell types**

**Table S7: Top 200 significantly up-regulated genes of dendritic cells in comparison to all other cell types**

**Table S8: Top 200 significantly up-regulated genes of lymphoid cells in comparison to all other cell types**

**Table S9: Top 200 mesenchymal cell genes up-regulated by EDS treatment**

**Table S10: Top 200 mesenchymal cell genes down-regulated by EDS treatment**

**‘**

**Table S11: Endothelial cell genes significantly affected by EDS treatment**

**
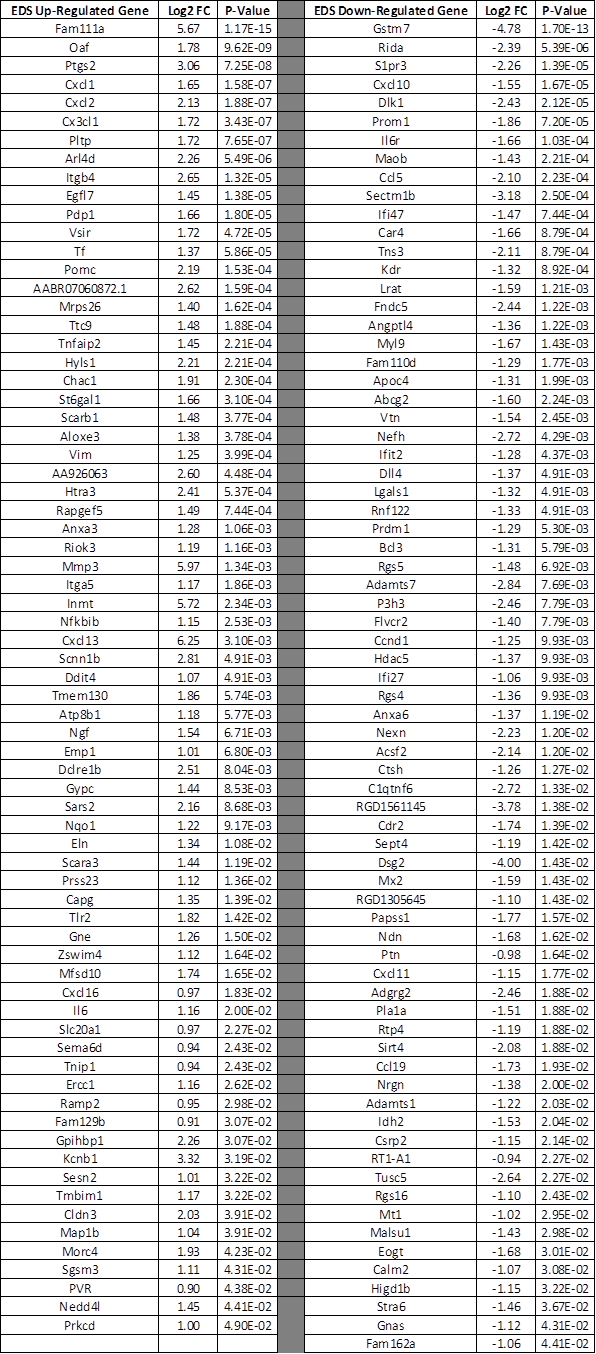
**

**Table S12: Smooth muscle cell genes significantly affected by EDS treatment**

**
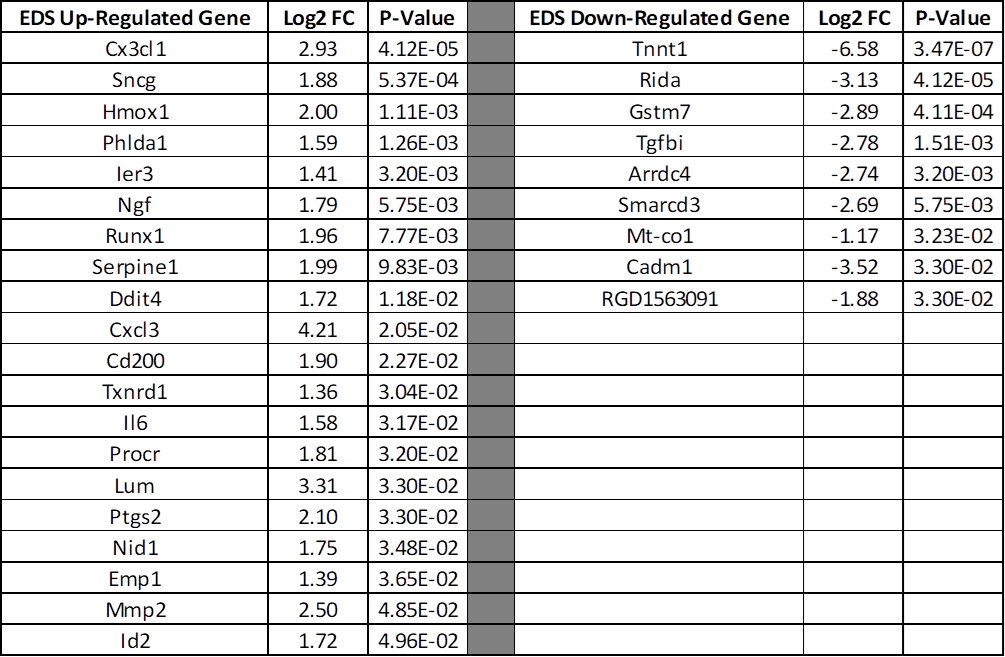
**

**Table S13: Top 100 significantly up-regulated genes of mesenchymal population one in comparison to all other populations**

**Table S14: Top 100 significantly up-regulated genes of mesenchymal population two in comparison to all other populations**

**Table S15: Top 100 significantly up-regulated genes of mesenchymal population three in comparison to all other populations**

**Table S16: Top 100 significantly up-regulated genes of mesenchymal population four in comparison to all other populations**

**Table S17: Top 100 significantly up-regulated genes of mesenchymal population one of EDS-treated animals in comparison to all other populations**

**Table S18: Top 100 significantly up-regulated genes of mesenchymal population two of EDS-treated animals in comparison to all other populations**

**Table S19: Top 100 significantly up-regulated genes of mesenchymal population three of EDS-treated animals in comparison to all other populations**

**Table S20: Top 100 significantly up-regulated genes of mesenchymal population four of EDS-treated animals in comparison to all other populations**

**Table S21: Top 100 significantly up-regulated genes of mesenchymal population five of EDS-treated animals in comparison to all other populations**

**Figure S1: Top 20 KEGG terms enriched for the 4 major cell types.**

**
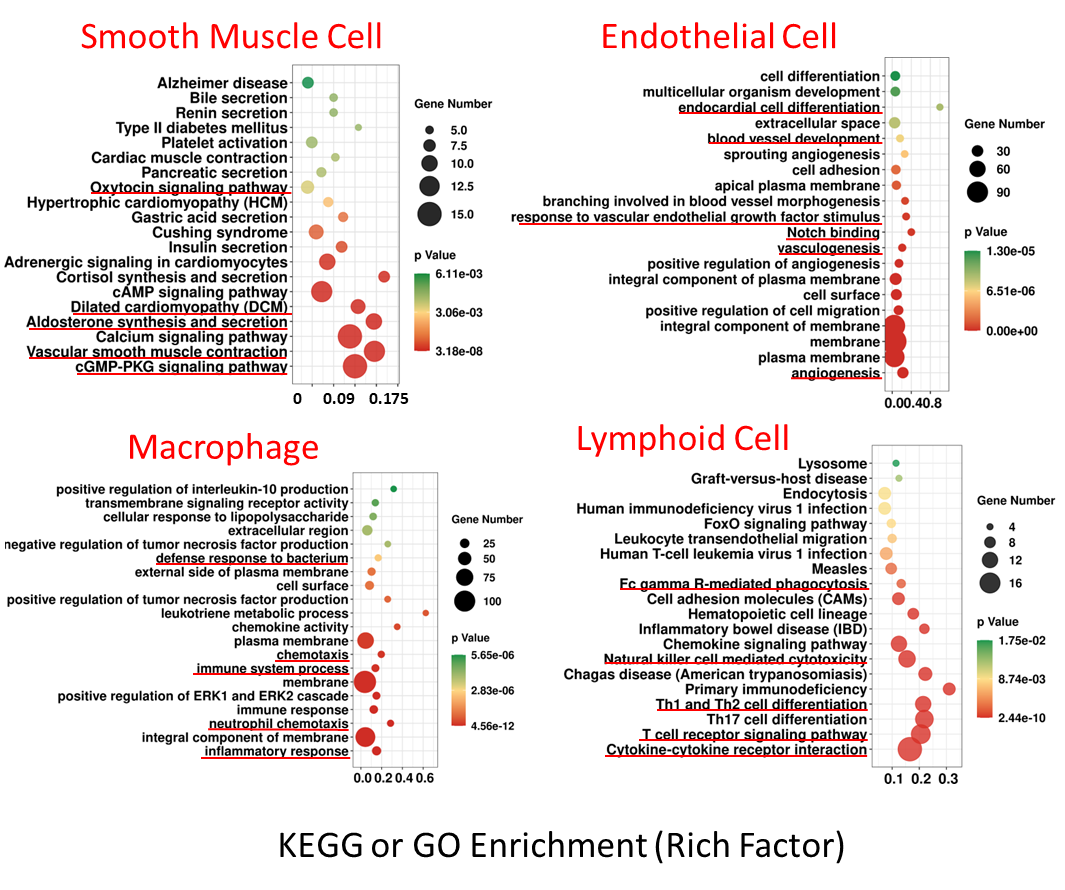
**

**Figure S2: Effect of EDS on mesenchymal cell transcriptome** (GO and KEGG enrichments of the all genes significantly affected by EDS treatment: EDS up-regulated genes: 1260; EDS down-regulated genes: 981**)**

**
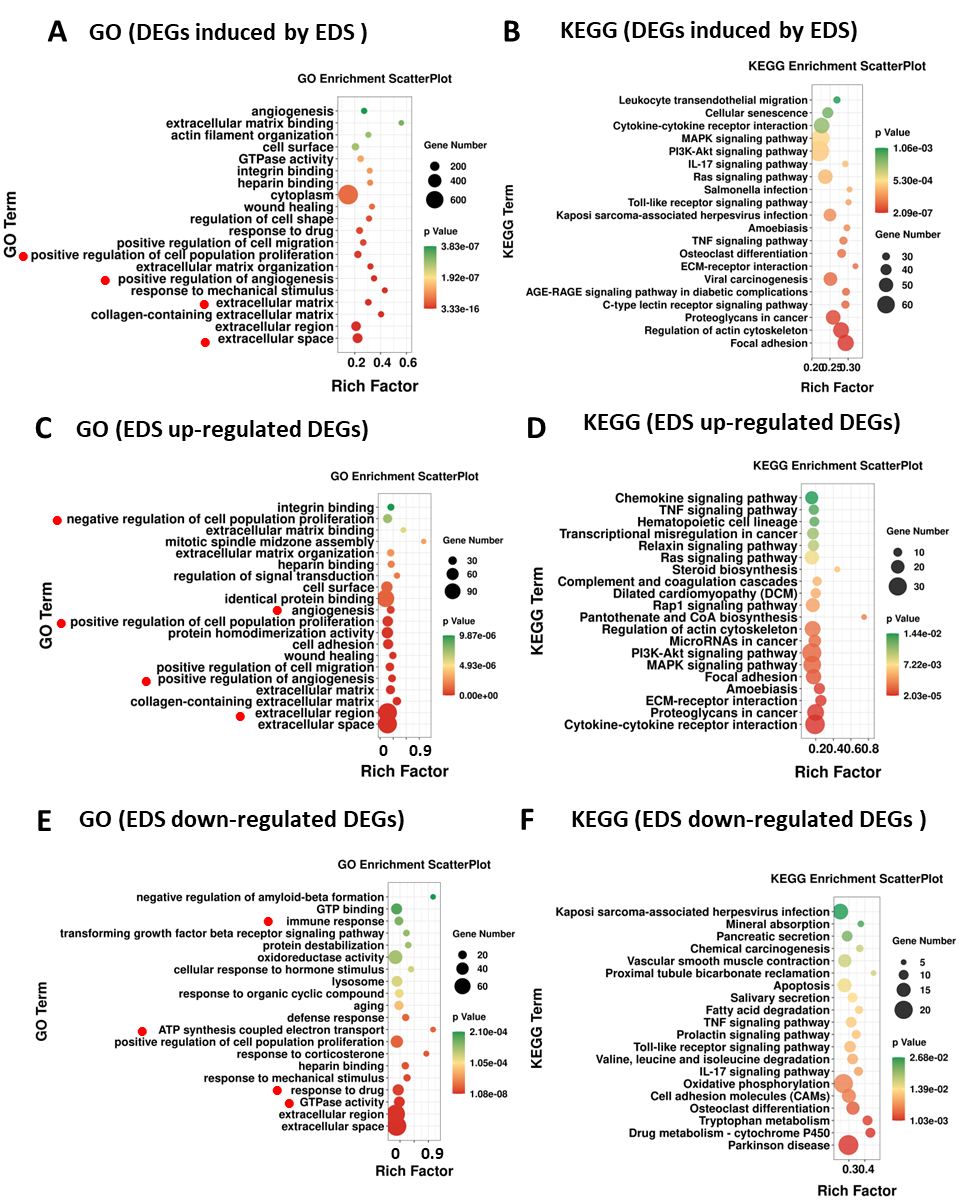
**

**●** Biological processes were discussed in the text.

**Figure S3: Effect of EDS on smooth muscle cell transcriptome** (GO and KEGG enrichments of the all genes significantly affected by EDS treatment: EDS up-regulated genes: 20; EDS down-regulated genes: 9**)**

**
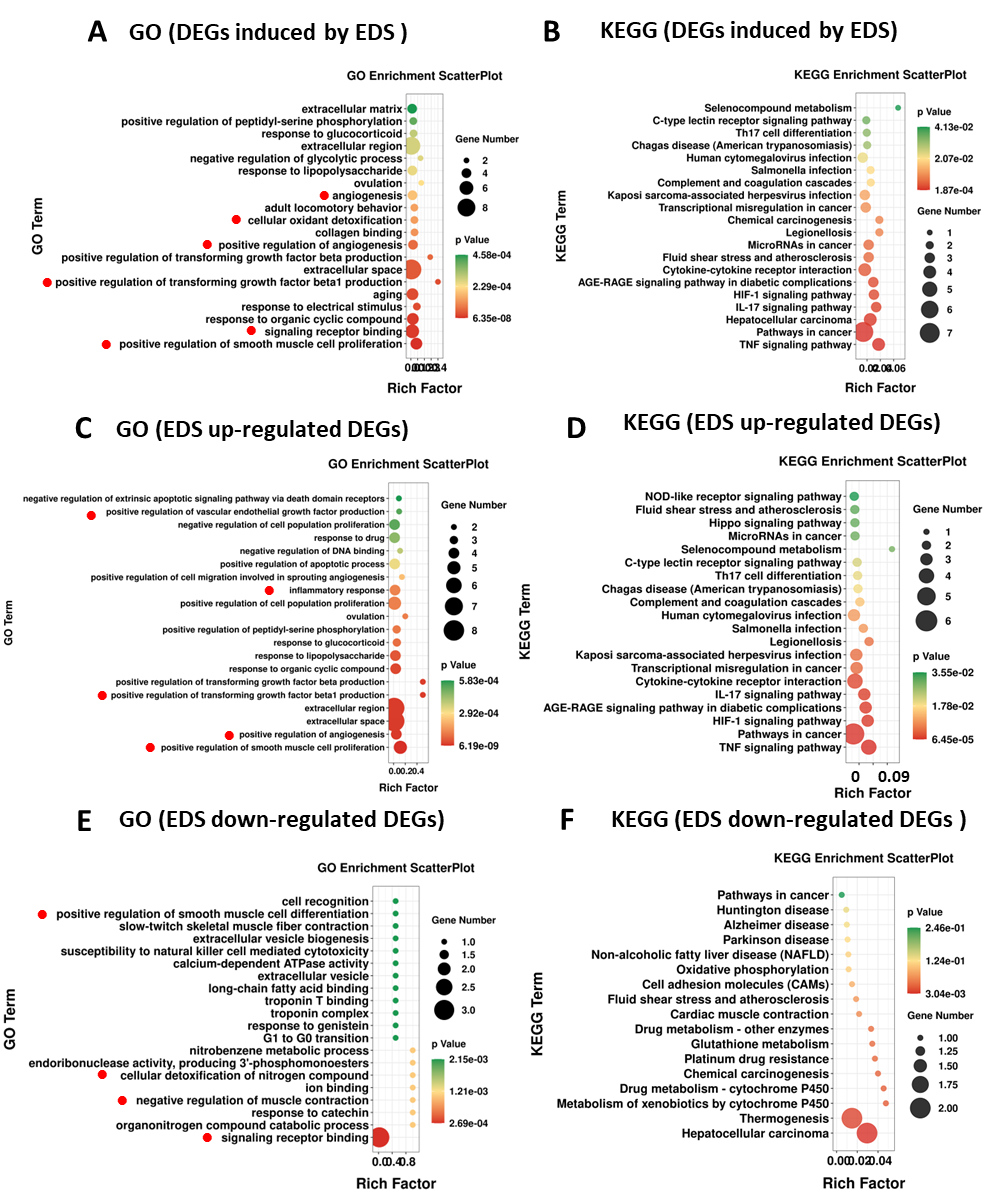
**

**●** Biological processes were discussed in the text.

**Figure S4: Effect of EDS on endothelial cell transcriptome** (GO and KEGG enrichments of the all genes significantly affected by EDS treatment: EDS up-regulated genes: 71; EDS down-regulated genes: 72**)**

**
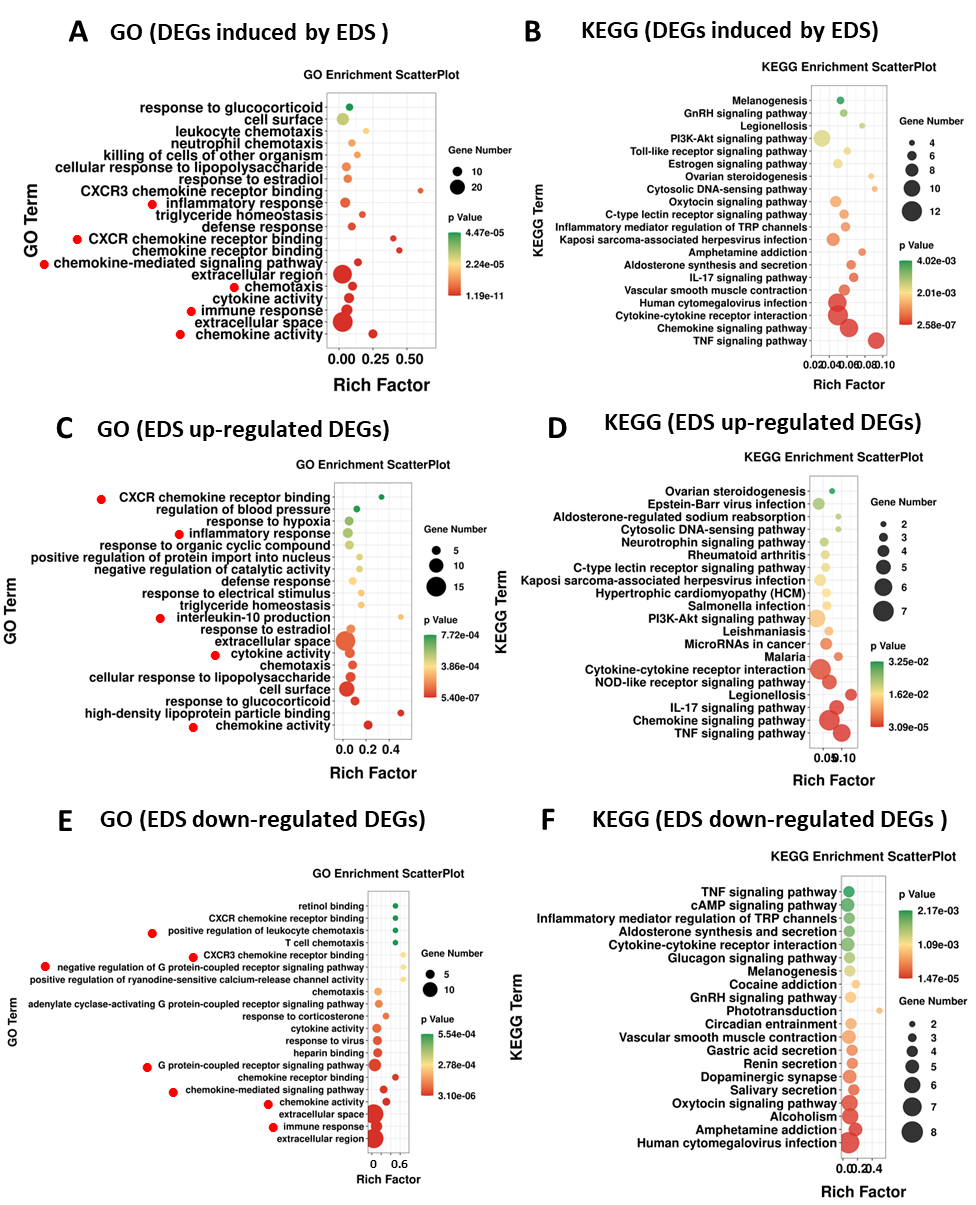
**

**●** Biological processes were discussed in the text.

**Figure S5: Effects of EDS (E1W) on interstitial cell numbers and proliferation**

**
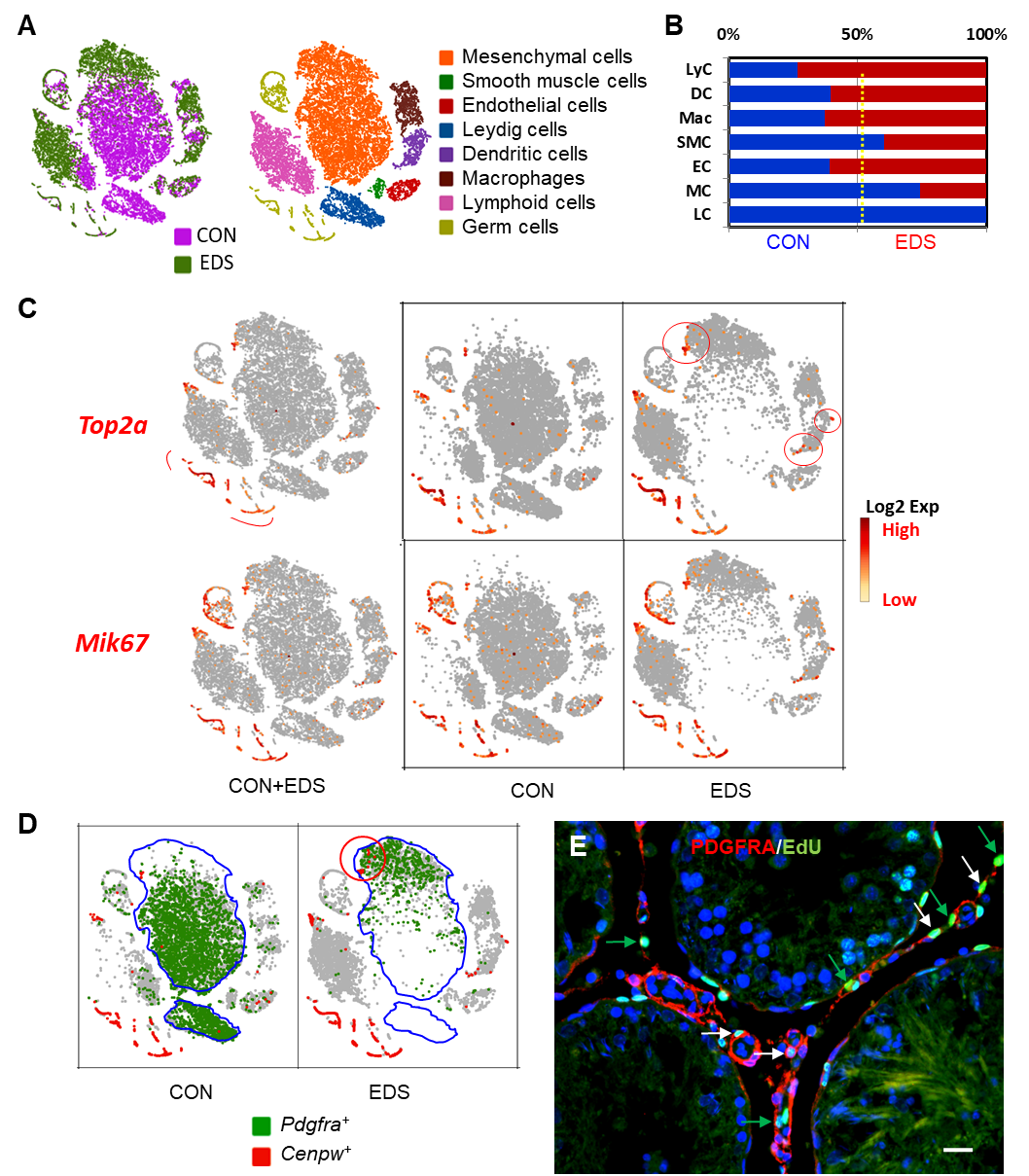
**

The Characterization of interstitial cells of CON (10,648 cells) and EDS treated (7,293 cells) samples is as follows:

(A) A tSNE plot shows the cell distributions of the two combined samples, identifying eight unique cell types.

(B) There is a cell number difference for each cell type between the two samples.

(C) Expressions of division-related genes (*Top2a, Mik67*) are shown, with the dividing fractions significantly affected by EDS highlighted by red circles.

(D) A division-related gene (*Cenpw*, red) and a mesenchymal cell marker gene (*Pdgfra*, green) are co-localized in CON and EDS samples.

(E) PDGFRA (red) and EdU (green) are co-stained to show the intensive proliferation of mesenchymal cells in the EDS sample. Nuclei are stained blue by DAPI. Green arrows indicate dividing PDGFRA- cells, while white arrows indicate dividing PDGFRA+ cells. CON is the control. The scale bars represent 25 μm in length.**Figure S6: Identification of interstitial cells of control rats by the specific protein markers.**

**
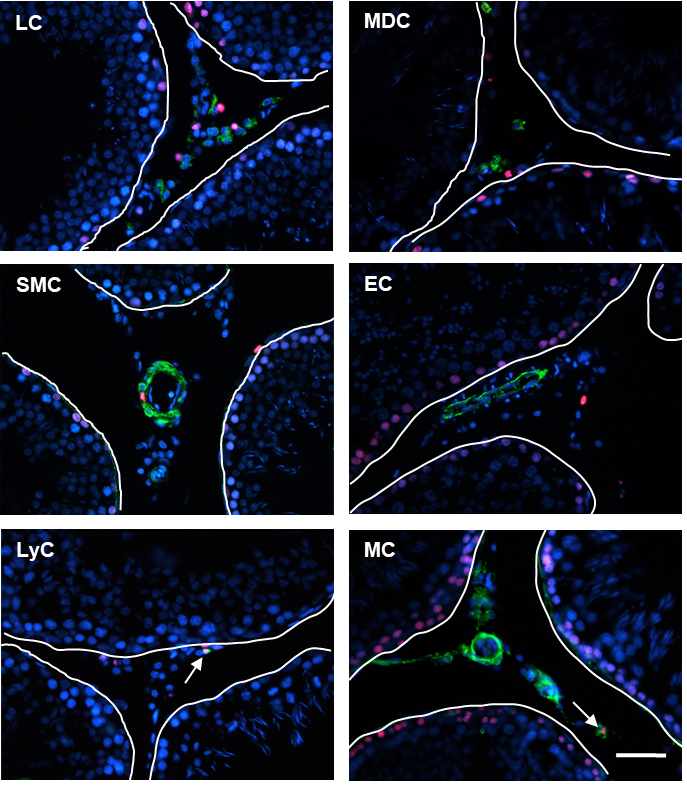
** The identification of interstitial cell types of control rats by specific protein markers is as follows. The seminiferous tubule boundaries are highlighted by white lines. LC: Leydig Cells (CYP17A1); MDC: Macrophages and Dendritic Cells (CD68); MC: Mesenchymal Cells (PDGFRA). SMC: Smooth Muscle Cells (ACTA2); EC: Endothelial Cells (CD31); LyC: Lymphoid Cells (CD3). Red: EdU; Green: Marker protein for each cell type. White arrow: Co-localizations of EdU and marker proteins. Representative photos of 4 control rats. Scale bars: 25 μm in length.

**Figure S7: Effect of EDS treatment on interstitial cell divisions**

**
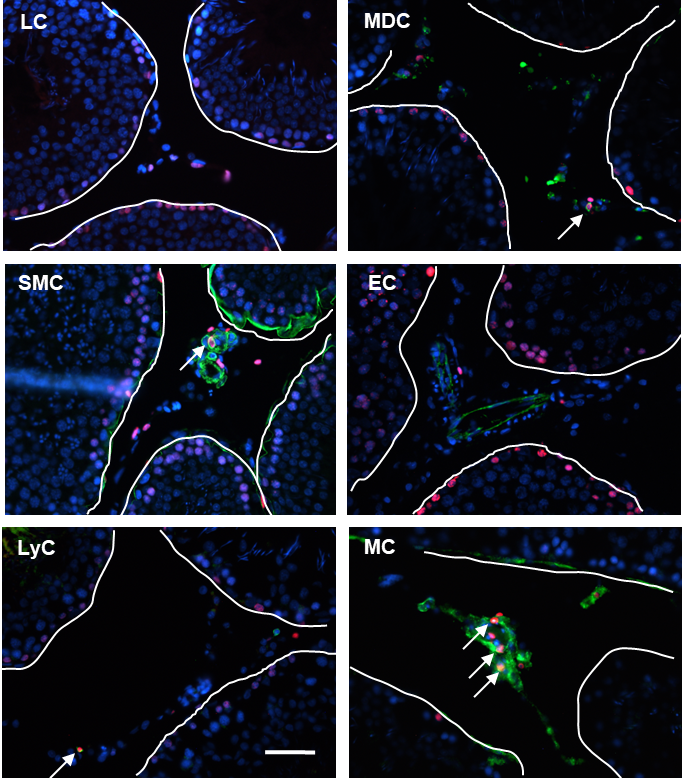
**

Identification of interstitial cell types of EDS-treated rats by the specific protein markers. The seminiferous tubule boundaries are highlighted by white lines. LC: Leydig Cells (CYP17A1); MDC: Macrophages and Dendritic Cells (CD68); MC: Mesenchymal Cells (PDGFRA). SMC: Smooth Muscle Cells (ACTA2); EC: Endothelial Cells (CD31); LyC: Lymphoid Cells (CD3). Red: EdU; Green: marker protein for each cell type. White arrow: Co-localizations of EdU and marker proteins. Representative photos of 4 EDS-treated rats. Scale bars: 25 μm in length.

**Figure S8: Effect of hemicastration on interstitial cell divisions**

**
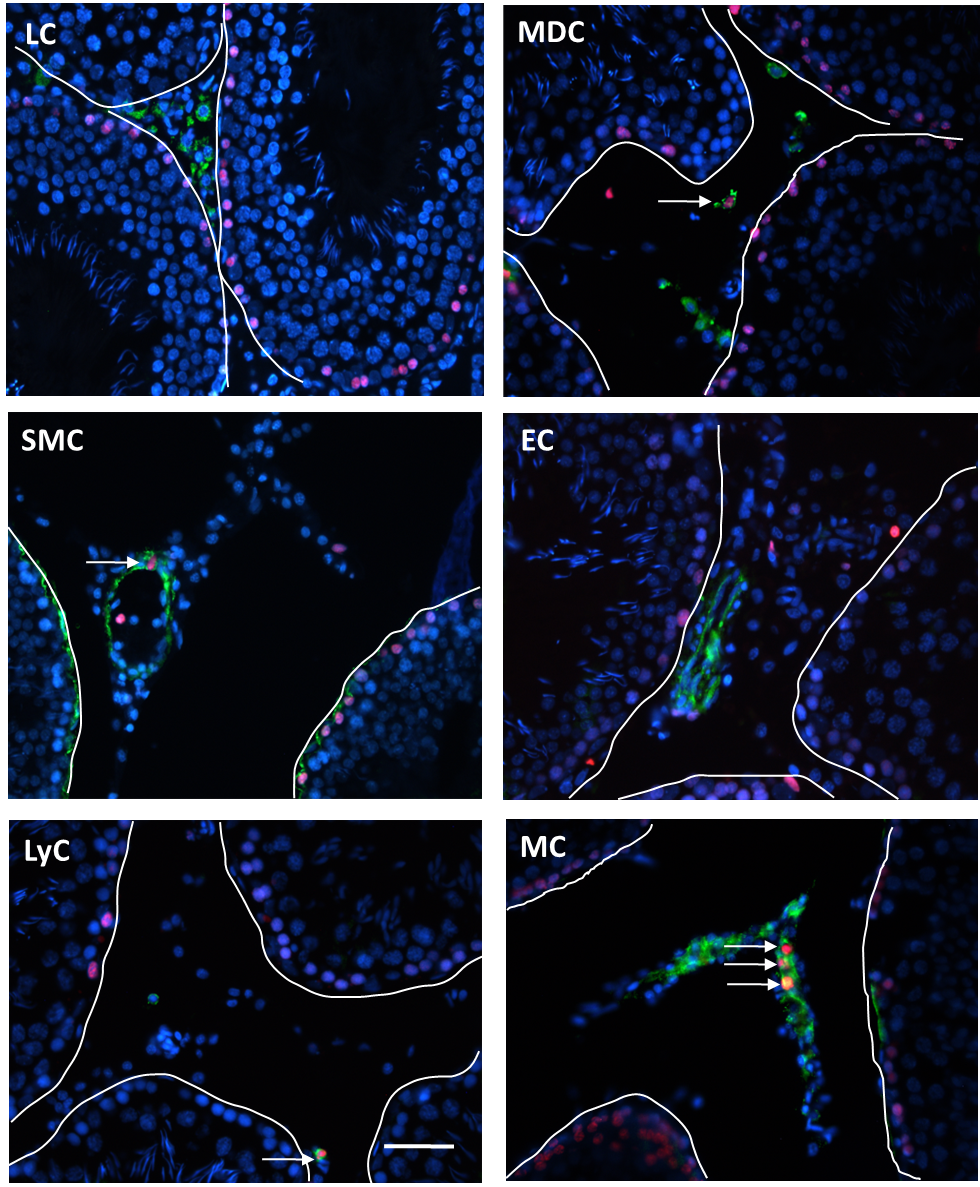
**


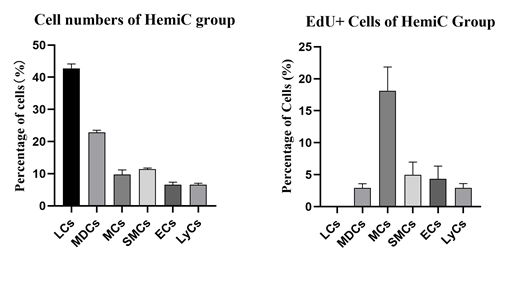
The quantification of interstitial cell types of hemicastrated rats by specific protein markers is presented. The boundaries of the seminiferous tubules are highlighted by white lines. LC: Leydig Cells (CYP17A1); MDC: Macrophages and Dendritic Cells (CD68); MC: Mesenchymal Cells (PDGFRA). SMC: Smooth Muscle Cells (ACTA2); EC: Endothelial Cells (CD31); LyC: Lymphoid Cells (CD3). Red: EdU; Green: Marker protein for each cell type. White arrow: Co-localizations of EdU and marker proteins. These are representative photos of 4 hemicastrated rats. The scale bars are 25 μm in length.

**Figure S9: Effect of EDS and hemicastration on serum testosterone (T) and marker protein contents.**


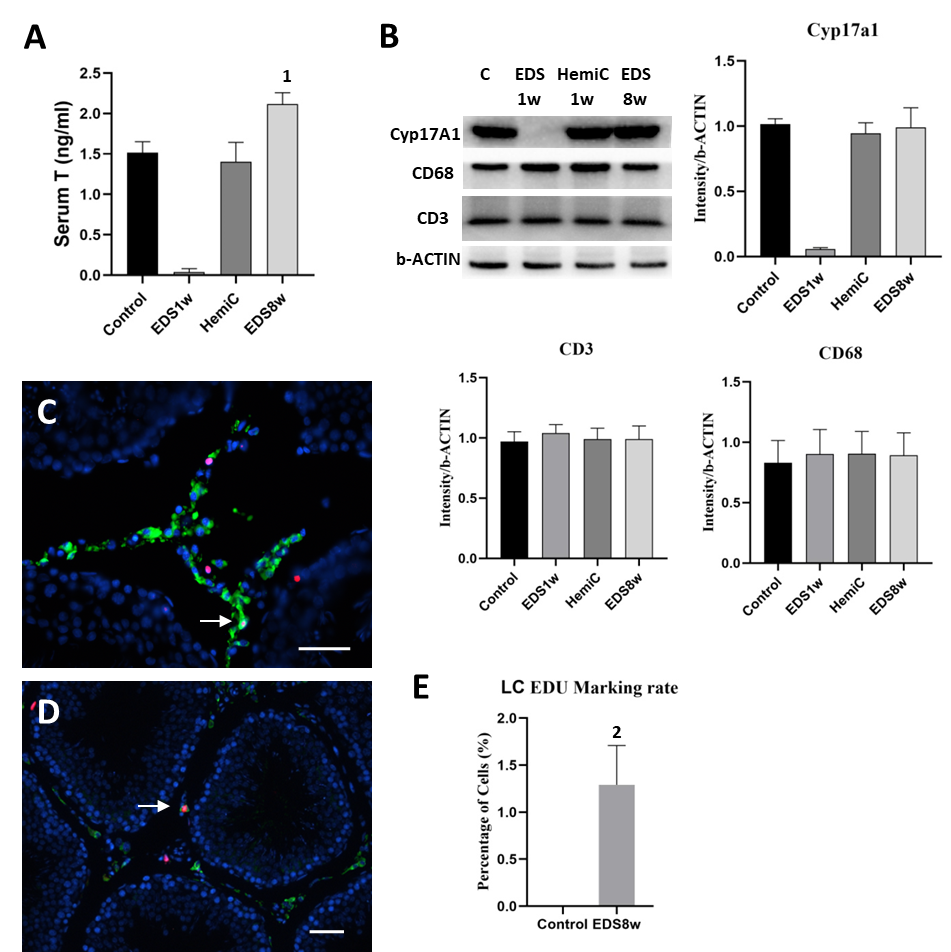


(A) Serum T concentrations.

(B) Western blot analysis and quantification of marker proteins for Leydig cells (CYP17A1), macrophages plus dendritic cells (CD68), and lymphoid cells (CD3).

(C) Co-staining of apoptotic nuclei (red) and Leydig cell marker (CYP17A1, green).

(D) Co-staining of EdU (red) and CYP17A1 (green) in an 8-week-EDS sample.

(E) Quantification of EdU^+^ cells within the CYP17A^+^ population of an 8-week-EDS sample. White arrows: double positive cells.

Data are expressed as the mean ± SE of 4 individual animals (n = 4).

1 Significantly different from all other groups with P < 0.05.

2 Significantly different from the control with P < 0.05.

ND: no detectable.

Scar bars represent 50 μm in length.

**Figure S10: Comparison of the top 10 DEGs among CON, E1W and E3W groups**

**
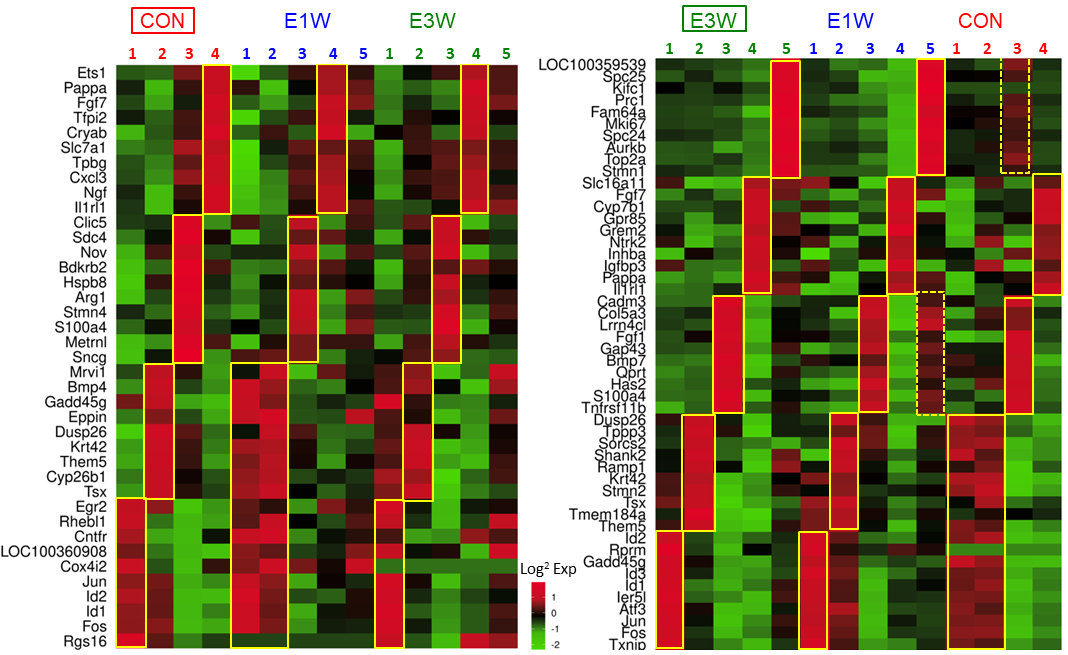
**

(Left) Expression of the top 10 DEGs of CON group by E1W and E3W groups.

(Right) Expression of the top 10 DEGs of E3W group by E1W and CON groups.

Boxes with broken line indicate that cluster 3 share more genes with cluster 5 (dividing cells).

**Figure S11: Top 20 GO terms enriched for the 4 clusters of control group.**

**
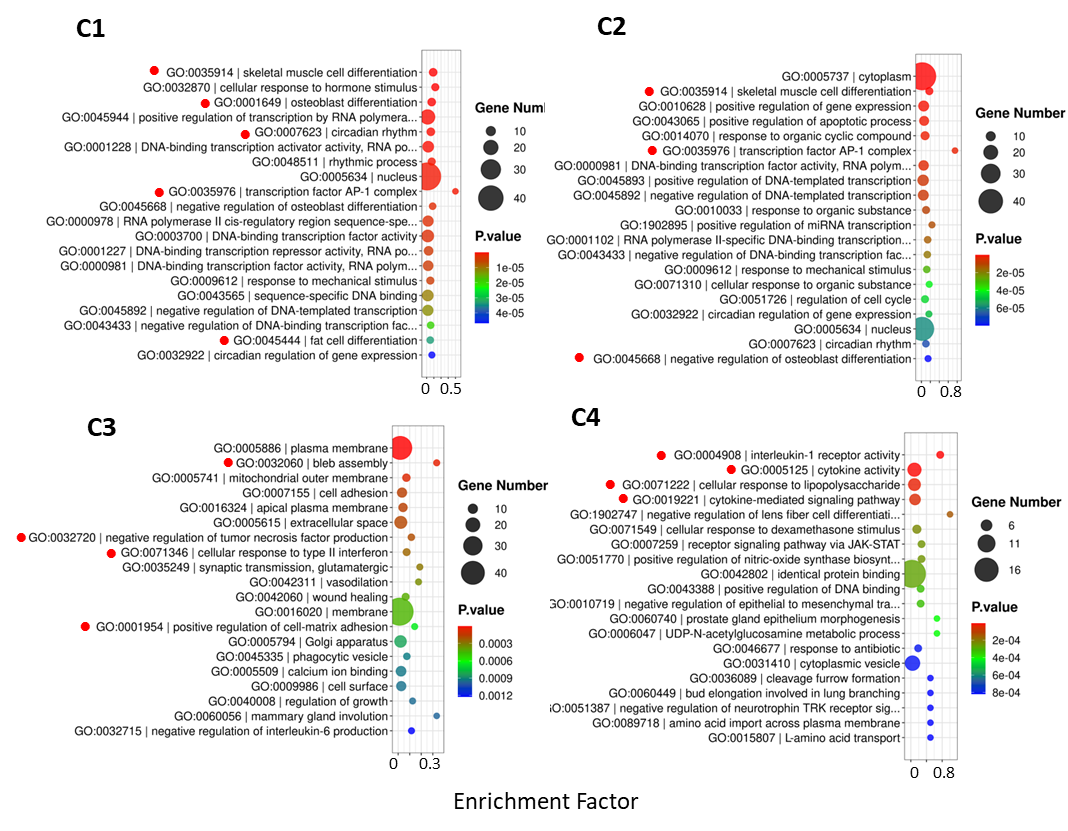
**

**●** Important functions defining the characteristic of the cells.

**Figure S12: Top 20 KEGG terms enriched for the 4 clusters of control group.**

**
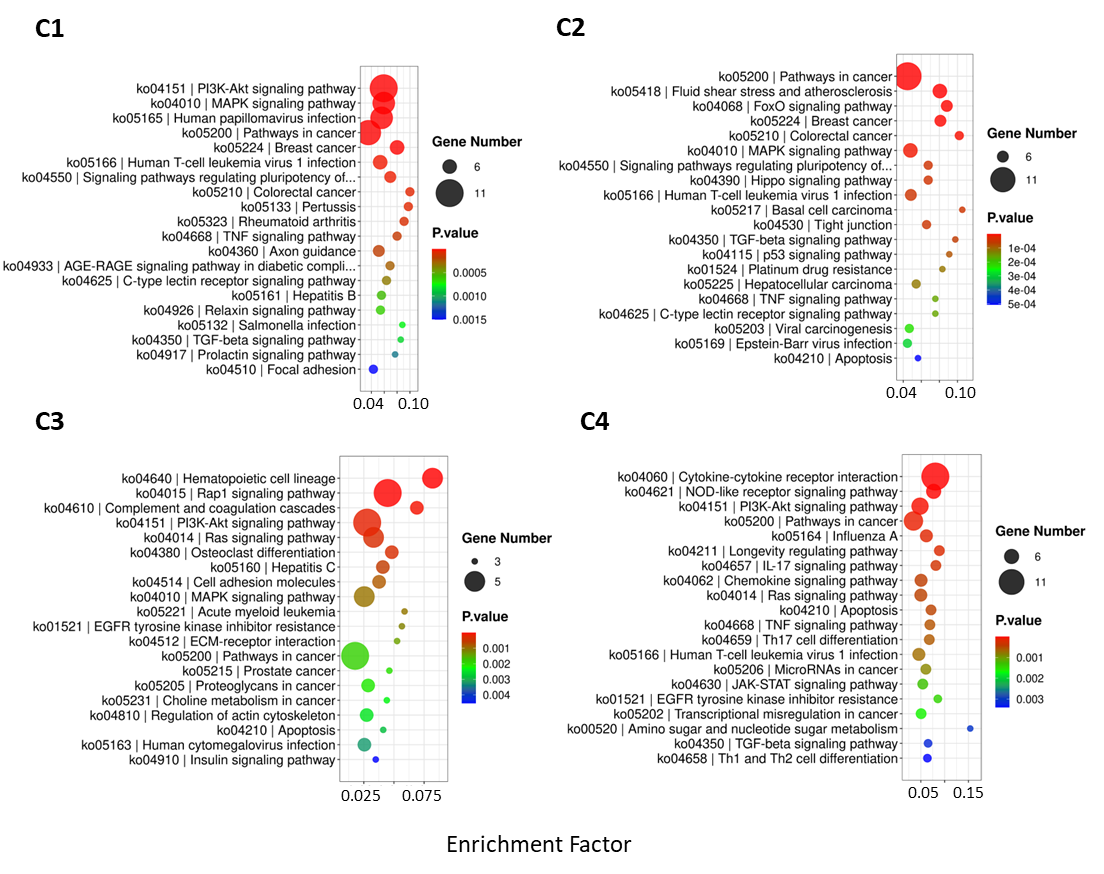
**

**Figure S13: Top 20 GO or KEGG terms enriched for the 5 clusters of E1W group.**

**
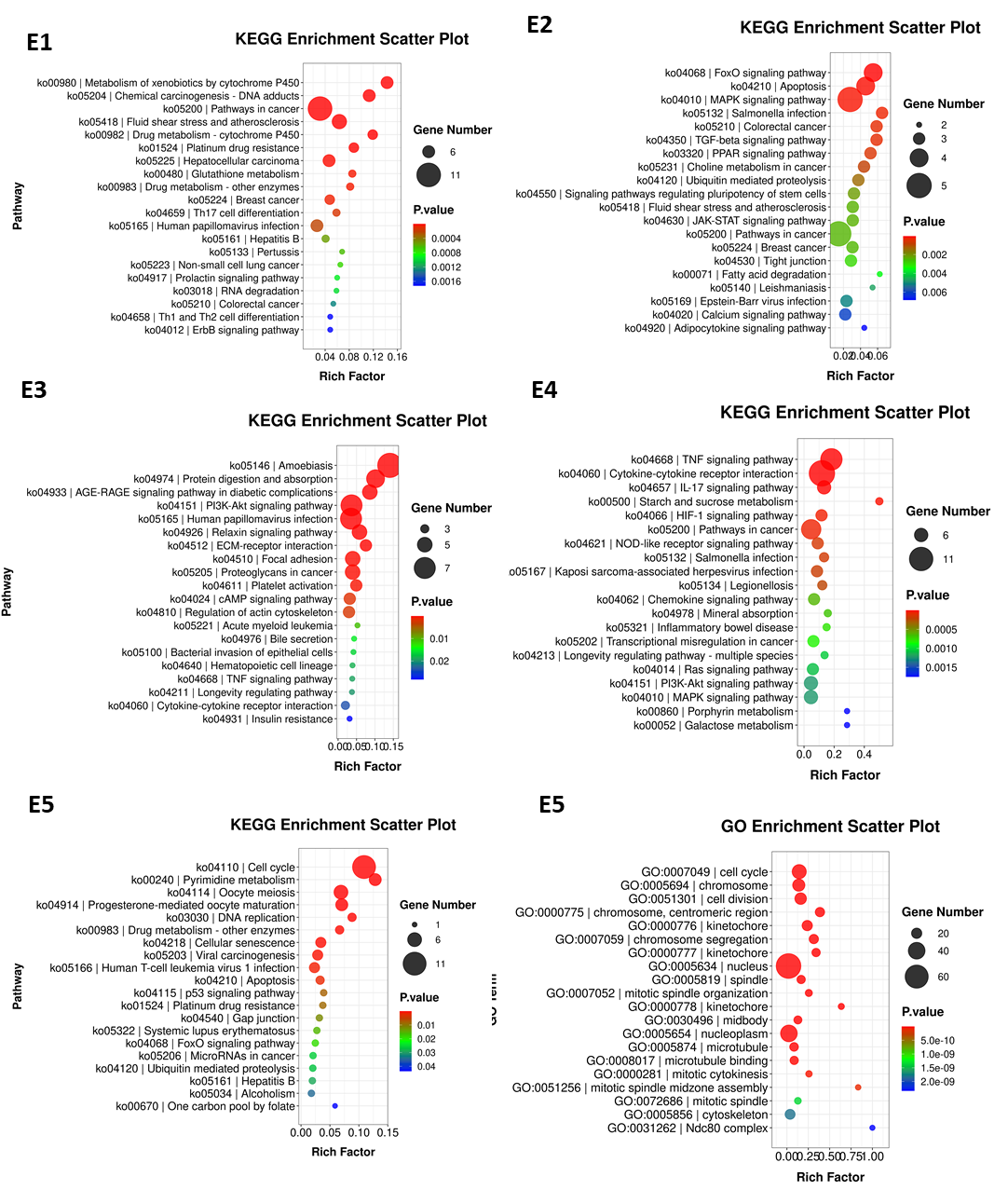
**

**Figure S14: Expression of SLC-enriched genes of CON group by E1W and E3W groups.**

**
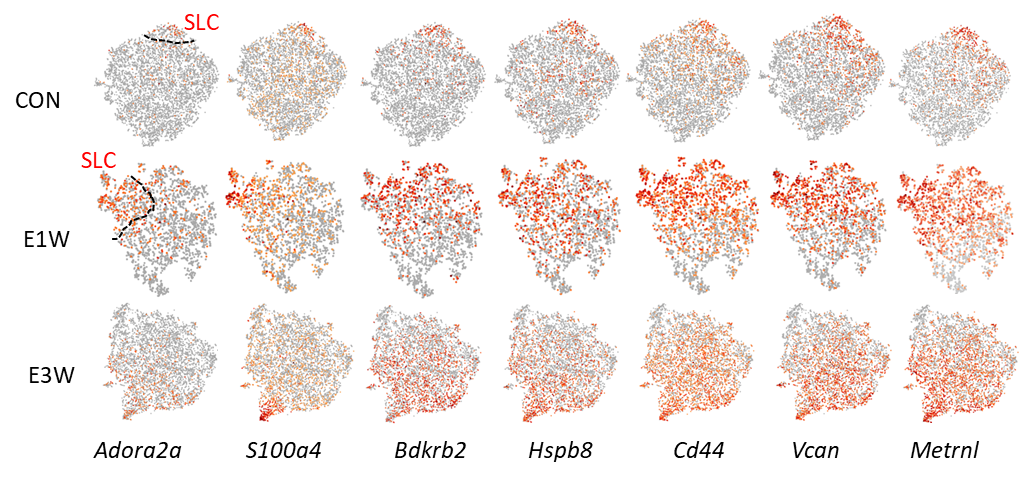
**

**Figure S15: Expression of ARG1 by potential SLCs of CON or E1W animals.**

**
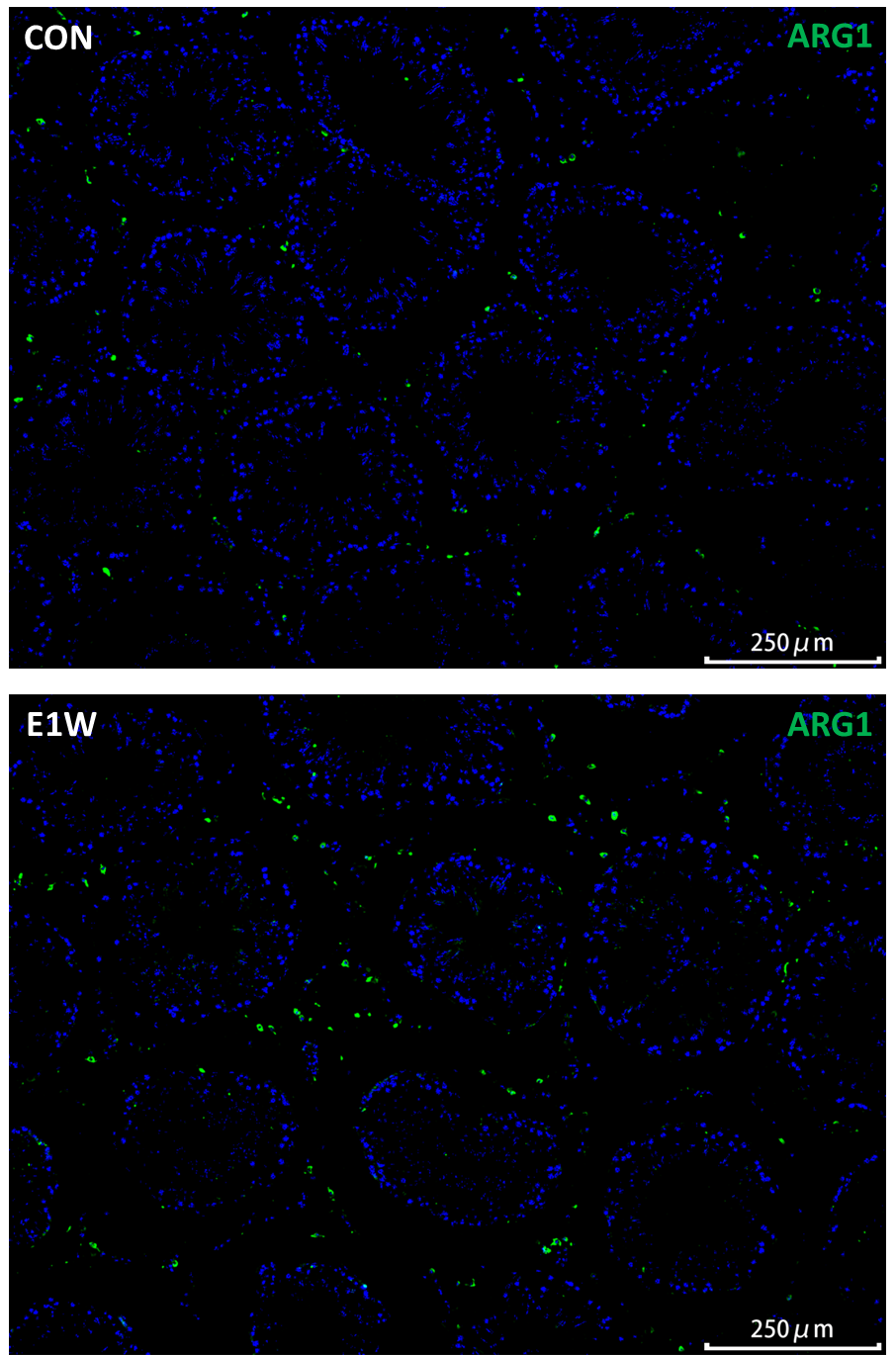
**

**Figure S16: Co-expression of ARG1 and CD68 by the interstitial cells of CON or E1W animals.**

**
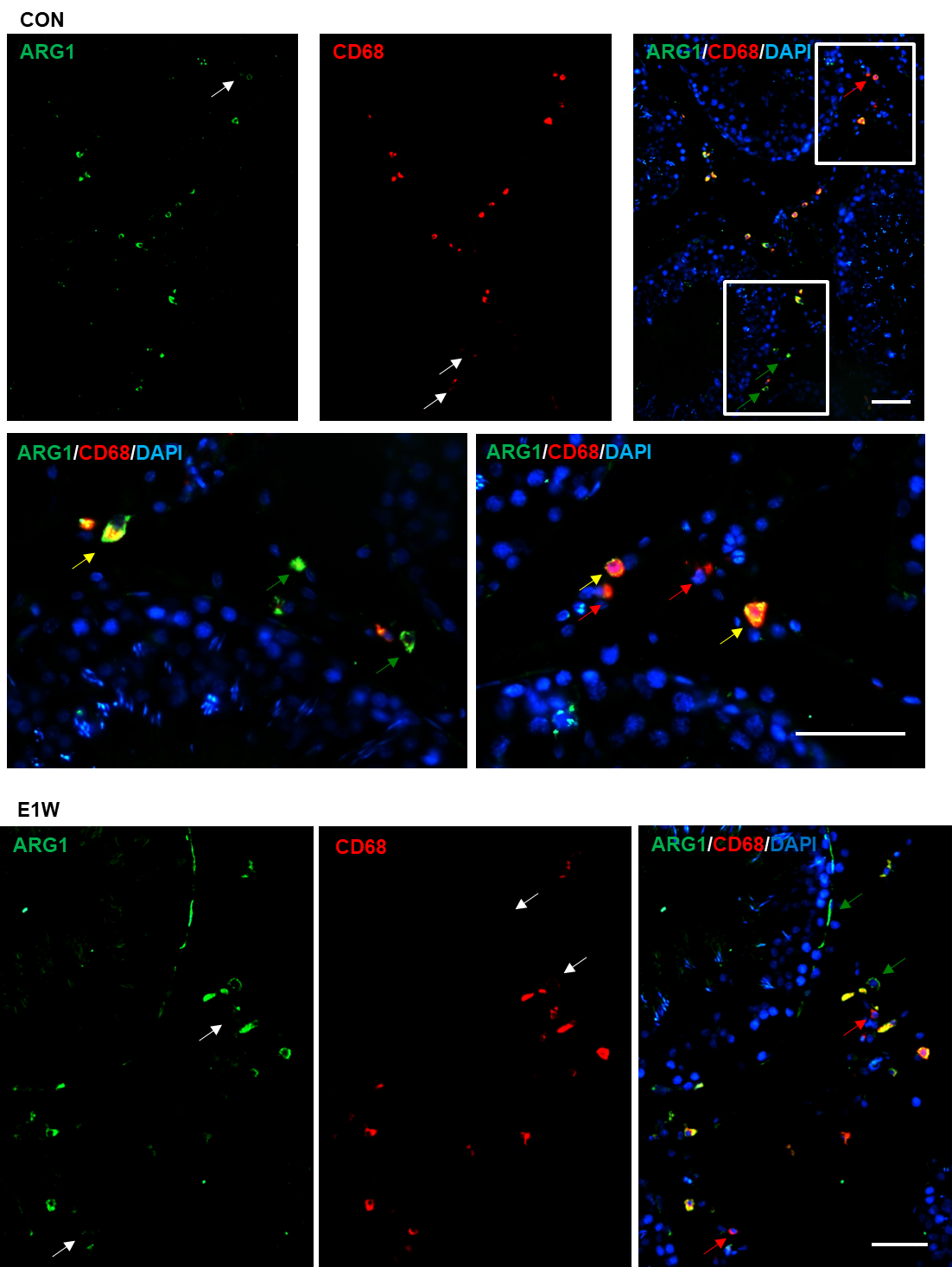
**

Green arrows: ARG^+^ /CD68^-^ cells; Red arrows: ARG^-^/CD68^+^ cells; Yellow arrows: ARG^+^/CD68^+^ cells; White arrows: where positive cells could be. Scale bar represents 100 um in length.

**Figure S17: Expression of GAP43 by potential SLCs of CON or E1W animals.**

**
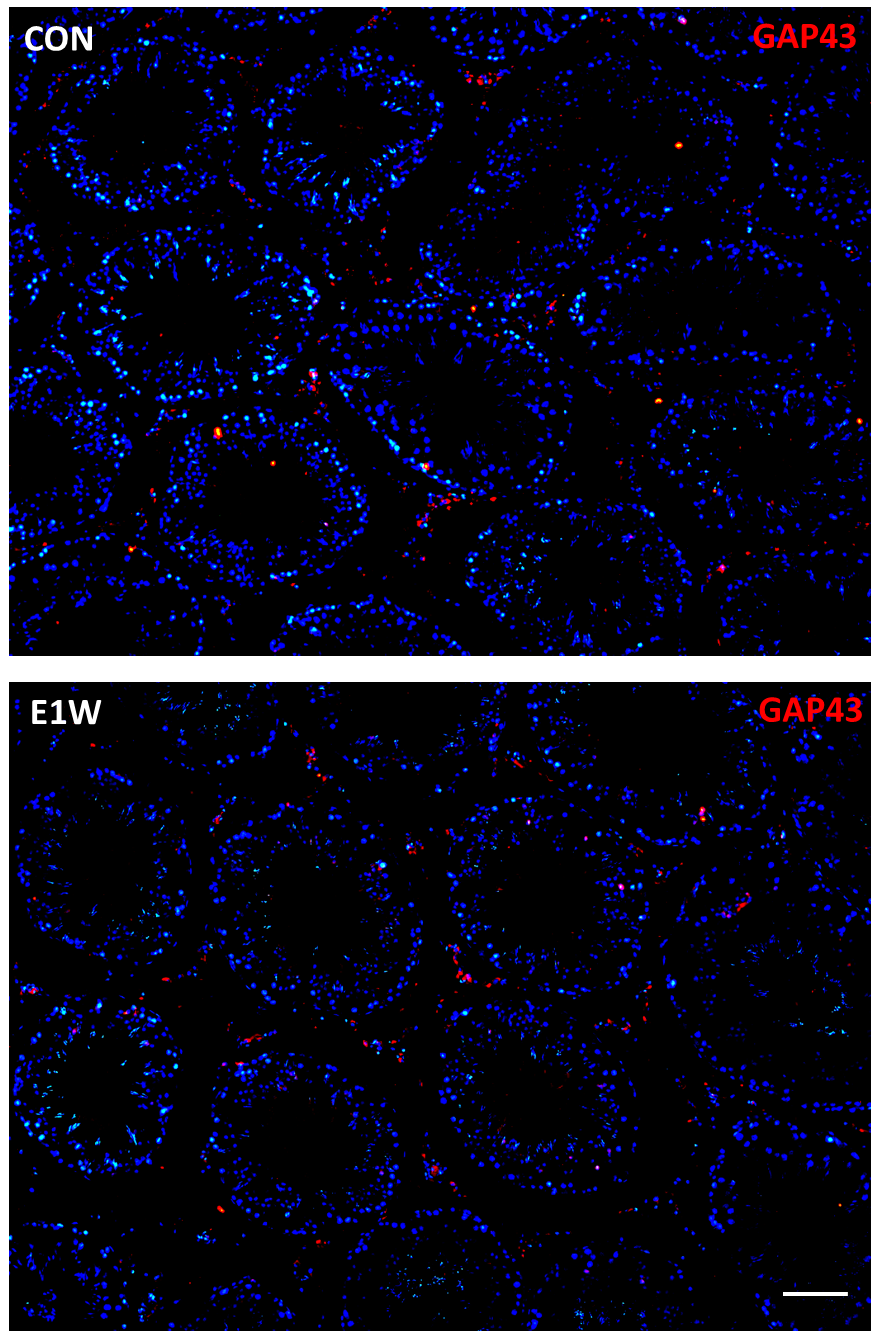
**

Scale bar represents 100 um in length.

**Figure S18: Expression of FN1 by interstitial cells of E1W animals.**

**
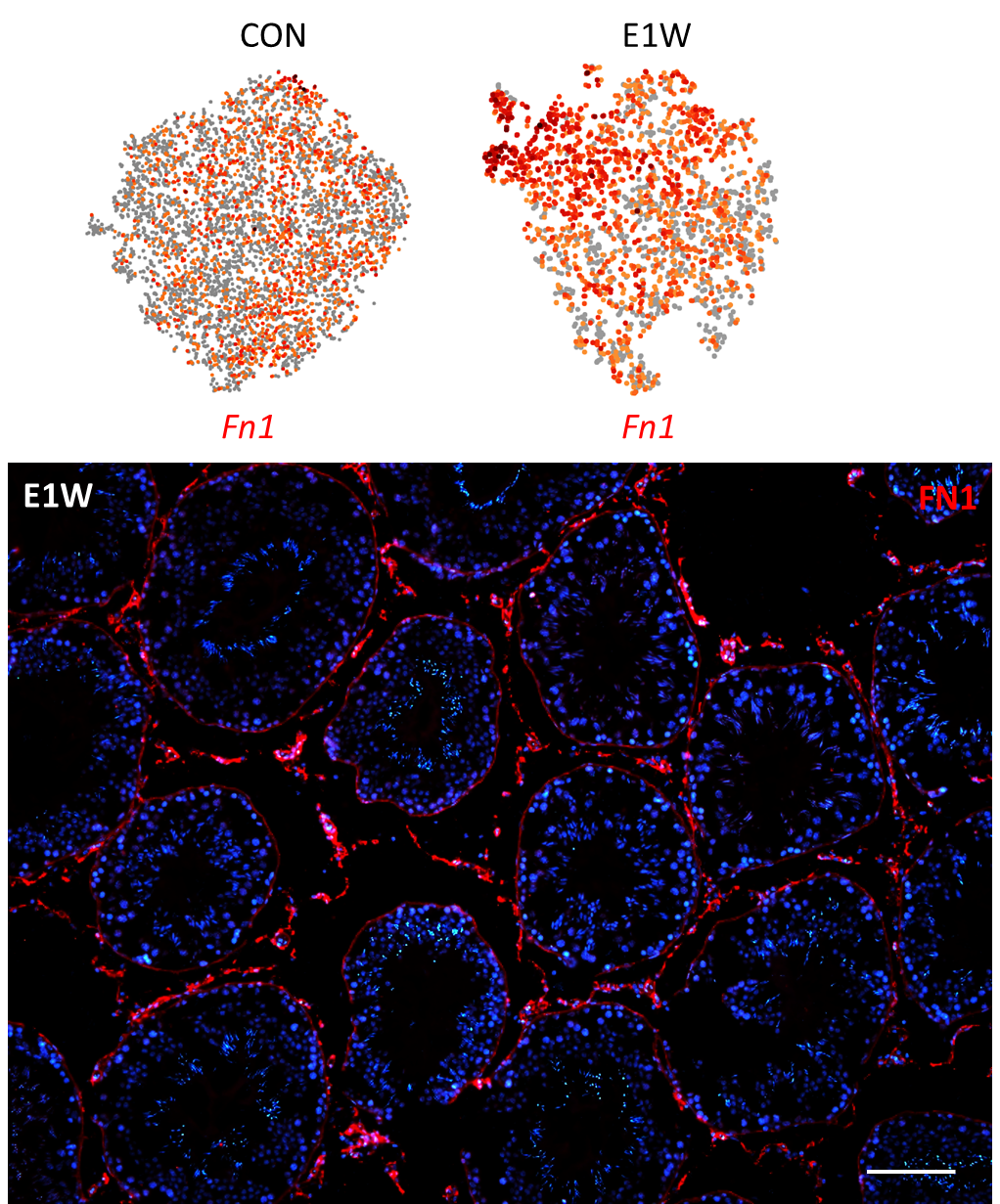
**

Scale bar represents 100 um in length.
